# ﻿Morphological and phylogenetic analyses reveal one new genus and six new species in *Irpicaceae* and *Steccherinaceae* (*Polyporales*, *Basidiomycota*) from the Yunnan–Guizhou Plateau, Asia

**DOI:** 10.3897/imafungus.16.172367

**Published:** 2025-12-29

**Authors:** Yinglian Deng, Meng Chen, Kaisheng Wang, Wanting Liu, Daxiang Chen, Shunqiang Yang, Wenli Li, Hongmin Zhou, Changlin Zhao

**Affiliations:** 1 College of Forestry, Key Laboratory of Forest Disaster Warning and Control in Universities of Yunnan Province, Southwest Forestry University, Kunming 650224, China; 2 College of Forestry, Southwest Forestry University, Kunming 650224, China; 3 Yunnan Tongbiguan Provincial Nature Reserve, Mangshi 679319, China; 4 Yunnan Key Laboratory of Gastrodia and Fungal Symbiotic Biology, Zhaotong University, Zhaotong 657000, China; 5 Modern Industry School of Edible-fungi, Southwest Forestry University, Kunming 650224, China; 6 Department Microbial Drugs (MWIS), Helmholtz-Centre for Infection Research, 38124 Braunschweig, Germany

**Keywords:** Molecular systematics, multi-gene phylogeny, new taxa, taxonomy, wood-inhabiting fungi

## Abstract

*Polyporales* is a diverse group of *Agaricomycetes* that has received extensive attention and study. Species in *Irpicaceae* and *Steccherinaceae (Polyporales)* are important wood-decaying fungi that cause white rot on both angiosperm and gymnosperm wood. Recently, many studies have focused on the classification of *Irpicaceae* and *Steccherinaceae*, but the familial placements of some taxa remain unresolved. In the present study, phylogenies of species in the two families were reconstructed using multilocus DNA sequence data, including ITS, nLSU, mtSSU, *tef*1-α, and *rpb*2, as well as two combined datasets: ITS+nLSU+mtSSU+*rpb*2+*tef*1-α for *Steccherinaceae* and ITS+nLSU for *Efibula* and related species. Three new species of *Efibula*, one new species of *Phanericium* within the family *Irpicaceae*, and one new genus, *Odentinium*, including two new species, *O. aurantium* and *O. cremeum*, within the family *Steccherinaceae*, are described and illustrated. The genus *Odentinium* is characterized by resupinate basidiomata with an odontioid hymenial surface, a monomitic hyphal system with clamped generative hyphae, cylindrical cystidia that are strongly encrusted, and ellipsoid, smooth basidiospores. Furthermore, the evolutionary times of *Polyporales*, including *Irpicaceae* and *Steccherinaceae*, were inferred based on conserved regions of DNA sequences (ITS+nLSU+mtSSU+*rpb*2+*tef*1-α). Bayesian evolutionary analysis indicated that the ancestors of *Irpicaceae* and *Steccherinaceae* are estimated to have emerged with mean crown ages of 199.17 Mya and 142.95 Mya, respectively, followed by the genus *Odentinium*, with a mean crown age of 120.8 Mya.

## ﻿Introduction

Fungi play a key role in many biological processes, influencing various complex environments, particularly across almost all ecosystems (Schimann et al. 2017; Luo et al. 2022; Jayawardena et al. 2023; Zhao et al. 2023a, b, c; Crous et al. 2024; Liu et al. 2025a, b; Wijesinghe et al. 2025). They are eukaryotic microorganisms that play key ecological roles as decomposers, saprobes, endophytes, epiphytes, and symbionts or pathogens of both animals and plants (Wu et al. 2016; Cui et al. 2018; Bhunjun et al. 2024; Qin et al. 2024; Chen et al. 2025). Fungi are a diverse and ecologically significant branch of the tree of life (James et al. 2020). A healthy ecosystem is composed of four components, namely non-biological components, producers, consumers, and decomposers, in which fungi play an extremely important role as decomposers by driving multiple material recycling processes in forest ecosystems (Wei and Dai 2004; Wang et al. 2013; Li et al. 2022; Hyde et al. 2023; Jayawardena et al. 2023; Wijesinghe et al. 2025). The value of fungi is indisputable, with both beneficial and negative impacts from a human perspective, acting as saprobes, epiphytes, endophytes, animal and plant pathogens, or symbionts (Chethana et al. 2021). Wood-decomposing fungi grow on various substrates such as living trees, dead standing trees, fallen trunks, branches and twigs, stumps, rotten wood, and soil (Lutzoni et al. 2004; Wu et al. 2014; M’Barek et al. 2020; Runnel et al. 2021; Wu et al. 2022a, b; Case et al. 2025; Cui et al. 2025a).

Due to advances in phylogenomics and increased taxon sampling, the higher-level relationships of the fungal tree of life are, for the most part, well resolved (Hibbett et al. 2025). Studies indicate that approximately 53,000 species have been described within Basidiomycota (Hibbett et al. 2025). Wood-inhabiting fungi are cosmopolitan and rich in diversity, growing in tropical, subtropical, temperate, and boreal regions, and exhibit diverse morphological features in their basidiomata (Dai 2010, 2011, 2012; Ryvarden and Melo 2014; Dai et al. 2021; Zhou et al. 2021, 2023; He et al. 2022; Zhang et al. 2023; Dong et al. 2024; Cui et al. 2025b; Wang et al. 2024; Yang et al. 2025). How basidiomata complexity evolved has interested mycologists for decades and has received considerable attention recently (Nagy et al. 2023). The order *Polyporales* Gäum includes a wide variety of basidiomata types and hymenophore configurations, including bracket-shaped, effused-resupinate, and stipitate forms with poroid, lamellate, labyrinthiform, or smooth hymenophores, and a few species produce shelf-like or flabellate clusters of overlapping sporomes (Binder et al. 2013). In addition, macroscopic and microscopic characters are variable and occur in several families of *Polyporales*. Variations and transitions among basidiomata types exist, and no morphological synapomorphy unites *Polyporales* (Binder et al. 2013; Kües and Navarro-González 2015; Nagy et al. 2018; Virágh et al. 2022).

*Polyporales* is one of the major orders of Basidiomycota (Kirk et al. 2008). Most species of *Polyporales* are saprotrophic wood-decay fungi that cause white or brown rot of wood and play a vital role in forest ecosystem degradation and nutrient cycling (Liu et al. 2023a, b, c; Yuan et al. 2023a, b). Moreover, some species of *Polyporales* are edible or medicinal fungi or forest pathogens (Dai and Yang 2008; Dai et al. 2009; Rajchenberg and Robledo 2013; An et al. 2015; Wu et al. 2019; Cui et al. 2023; Ghobad-Nejhad et al. 2024; Yang et al. 2025). Due to their important ecological functions and economic value, *Polyporales* has been extensively studied, and its members have increased rapidly. Kirk et al. (2008) reported approximately 1,800 species, 216 genera, and 13 families in *Polyporales*, whereas by 2024, about 2,544 species, 285 genera, and 18 families had been recognized (He et al. 2024).

Previously, the establishment of families within *Polyporales* was primarily based on morphological characteristics. *Polyporaceae* Fr. ex Corda is the oldest family in *Polyporales* and was proposed by Fries (1838) to include most wood-inhabiting fungi with a poroid hymenophore. Subsequently, *Climacocystaceae* B.K. Cui, Shun Liu & Y.C. Dai, *Gloeoporellaceae* B.K. Cui, Shun Liu & Y.C. Dai, *Hypochniciaceae* J.H. Dong & C.L. Zhao, *Irpicaceae* Spirin and Zmitr., *Meruliaceae* Rea, *Neohypochniciaceae* J.H. Dong & C.L. Zhao, *Podoscyphaceae* D.A. Reid, *Sparassidaceae* Herter, and *Steccherinaceae* Parmasto were proposed successively and are currently recognized within *Polyporales* (Herter 1910; Rea 1922; Reid 1965; Parmasto 1968; Spirin 2003; Zhao et al. 2015; Justo et al. 2017; Liu et al. 2023b; Mao et al. 2023; Dong et al. 2024). *Polyporales* constitutes a highly diverse group, not only in molecular sequences but also in morphological characteristics, including basidiomata that are resupinate, effused-reflexed, pileate-sessile, pileate-stipitate, or cauliflower-like, and hymenophores that are poroid, daedaleoid, hydnoid, lamellate, labyrinthine, or odontoid. These fungi often form leathery, corky, or woody basidiomata that decay slowly. Although no strict morphological definition exists for the order, most polypore fungi can be readily recognized as members of *Polyporales* (Liu et al. 2023a, b, 2025a, b; He et al. 2024).

*Irpicaceae*, typified by *Irpex* Fr., was established by Spirin (2003). It belongs to the phlebioid clade within *Polyporales* and includes fourteen genera, viz., *Byssomerulius* Parmasto, *Ceriporia* Donk, *Crystallicutis* El-Gharabawy, *Cytidiella* Pouzar, *Efibula* Sheng H. Wu, Leal-Dutra & G.W. Griff., *Gloeoporus* Mont., *Irpex*, *Leptoporus* Quél., *Meruliopsis* Bondartsev, *Phanericium* (Parmasto) K.H. Larss. & Spirin, *Phanerochaetella* C.C. Chen & Sheng H. Wu, *Raduliporus* Spirin & Zmitr., *Resiniporus* Zmitr., and *Trametopsis* Tomšovský, with variable hymenophores, including corticioid and irpicoid species, and resupinate to pileate polypores (Chen et al. 2018, 2021, 2022; Larsson et al. 2025). Previous phylogenetic studies determined that *Efibula* was not recovered as monophyletic but formed three distinct clades (Floudas and Hibbett 2015; Chen et al. 2021; Li et al. 2022), one of which was described as the new genus *Phanericium* (Parmasto) K.H. Larss. & Spirin by Larsson et al. (2025). Meanwhile, four species, *Efibula americana* Floudas & Hibbett, *E. rodriguezarmasiae* Tellería, M. Dueñas, Beltrán-Tej., Melo, Salcedo & M.P. Martín, *E. subglobispora* C.C. Chen & Sheng H. Wu, and *E. taiwanensis* Yue Li & S.H. He, were proposed to be combined into the new genus *Phanericium* based on similar morphology and correlative phylogeny (Larsson et al. 2025).

*Steccherinaceae* was typified by the genus *Steccherinum* Gray (1968). It belongs to the residual polyporoid clade of *Polyporales* (Basidiomycota). The family *Steccherinaceae*, in its original concept, included genera with smooth, hydnoid, or poroid hymenophores and was microscopically characterized by a dimitic hyphal system, generative hyphae with clamp connections, and a variable presence of cystidia (Parmasto 1968; Maas Geesteranus 1971). With the addition of molecular data, some authors used broad treatments of *Steccherinaceae*, and consequently, some genera were transferred to *Meruliaceae* (Larsson 2007; Zmitrovich 2018). Miettinen et al. (2012) used a multigene phylogenetic analysis that redefined *Steccherinaceae* beyond the characteristics traditionally known for the family and included genera with a monomitic hyphal system and generative hyphae with simple septa. Later, some overviews of families of *Polyporales* recovered *Steccherinaceae* as a monophyletic group nested in the residual polyporoid clade (Justo et al. 2017; Westphalen et al. 2021).

Comparative analyses of nucleotide sequences have concluded that molecular evolution occurs at a relatively constant rate, thereby giving rise to the concept of the molecular clock (Zuckerkandl 1962; Zuckerkandl and Pauling 1965). By using fossil records or geological events as calibration points in conjunction with phylogenetic trees as the foundational framework, estimation of divergence times among different taxonomic groups has become a widely adopted methodology in evolutionary biology (Avise and Johns 1999; Yang et al. 2007; Havill et al. 2008; Lockwood et al. 2013; Zhao et al. 2016, 2017; Varga et al. 2019; Ji et al. 2022; Zhao et al. 2024a, b). Molecular divergence times of fungi should reflect their different taxonomic ranks according to the geological ages at which they evolved (Hennig 1966; Zhao et al. 2016). Wang et al. (2023, 2025) clarified the taxonomy and phylogeny of *Ceriporia* and other related taxa in *Irpicaceae* using more specimens from around the world, especially from China, amended their definitions, studied morphologically confusing species, and speculated on the divergence time of *Irpicaceae*. The family *Steccherinaceae*, as a monophyletic group in *Polyporales*, included twenty-four genera (He et al. 2024). Although phylogenetic and morphological analyses of *Steccherinaceae* have been carried out (He et al. 2024), more relevant specimens are continuously being collected in China, and there is a lack of research on the divergence time of *Steccherinaceae*. In this study, the morphology, phylogeny, and divergence times of *Irpicaceae* and *Steccherinaceae* are investigated. In addition, three new species of *Efibula*, one new species of *Phanericium*, and one new genus, *Odentinium*, of *Steccherinaceae* are described and illustrated.

## ﻿Materials and methods

### ﻿Morphological studies

Fresh basidiomata of fungi growing on angiosperm branches in China were photographed in situ from July 2019 to January 2024. After the collection information was recorded (Rathnayaka et al. 2024), the specimens were taken to the laboratory and dried in an electric food dehydrator at 30–50 °C (Hu et al. 2022), then sealed and stored in envelope bags and deposited in the herbarium of Southwest Forestry University (SWFC), Kunming, Yunnan Province, P.R. China. Macromorphological descriptions were based on field notes and photographs captured in the field and laboratory. Color terminology followed Petersen (1996) and was confirmed in general terms according to the CMYK color code (Deep White Printing Team 2022).

Micromorphological data were obtained from dried specimens observed under a light microscope (Nikon Ni, Tokyo, Japan), following previous studies (Zhao et al. 2023a; Yang et al. 2025). The following abbreviations are used: KOH = 5% potassium hydroxide water solution, CB = Cotton Blue, CB– = acyanophilous, IKI = Melzer’s reagent, IKI– = both inamyloid and indextrinoid, L = mean spore length (arithmetic average of measured spores), W = mean spore width (arithmetic average of measured spores), Q = variation in the L/W ratios between the specimens studied, and n = a/b (number of spores (a) measured from a given number (b) of specimens). Standardized sampling of microstructures for measurement included basidiospores (30), basidia, basidioles, and cystidia (5), and hyphal diameters (10) (Dong et al. 2024; Yang et al. 2025).

### ﻿DNA extraction, PCR amplification, and sequencing

The EZNA HP Fungal DNA Kit (Omega Biotechnologies Co., Ltd., Kunming, China) was used to extract DNA from dried specimens with minor modifications. DNA samples were stored at –20 °C. The amplified fragments included the internal transcribed spacer ITS (ITS5 and ITS4), the large subunit nuclear ribosomal RNA gene nLSU (LR0R and LR7), the small subunit mitochondrial rRNA gene mtSSU (MS1 and MS2), the translation elongation factor 1-α gene *tef*1-α (ef1-983 F and ef1-2218R), and the second subunit of RNA polymerase II *rpb*2 (*brpb*2-6F and *brpb*2-7.1R) (White et al. 1990; Liu et al. 1999; Matheny et al. 2002; Matheny 2005; Rehner and Buckley 2005).

The PCR protocol for ITS and mtSSU consisted of an initial denaturation at 95 °C for 3 min, followed by 35 cycles at 94 °C for 40 s, 58 °C for ITS and 55 °C for mtSSU for 45 s, and 72 °C for 1 min, with a final extension at 72 °C for 10 min. The PCR protocol for nLSU and *tef*1-α consisted of an initial denaturation at 94 °C for 1 min, followed by 35 cycles at 94 °C for 30 s, 48 °C for nLSU and 59 °C for *tef*1-α for 1 min, and 72 °C for 1.5 min, with a final extension at 72 °C for 10 min. The PCR procedure for *rpb*2 consisted of an initial denaturation at 95 °C for 2.5 min, followed by 40 cycles at 95 °C for 30 s, 52 °C for 1 min, and 72 °C for 1 min, followed by an extension at 72 °C for 1.5 min, and a final extension at 72 °C for 5 min. Each 30 μL PCR reaction mixture contained 12.5 μL of double-distilled water, 15 μL of PCR Master Mix (Sangon Biotech Shanghai Co., Ltd.), 1 μL of each primer, and 1 μL of template DNA. Amplification followed the protocol of Dong et al. (2024). PCR products were examined using 1.5% agarose gel electrophoresis stained with GoldenView and sent to Qingke Co., China, for sequencing. Purification and sequencing were performed at Kunming Tsingke Biological Technology Limited Company, Kunming, Yunnan Province, P.R. China. Sequences were reviewed and manually edited using Chromas v.1.0.1.1 to remove low-quality base calls from both ends. All newly generated sequences were deposited in GenBank (Table 1).

**Table 1.** List of species, specimens, and GenBank accession numbers of sequences used in this study. [* Indicates the type of materials].

Species name	Specimen no.	GenBank accessions no					References
		ITS	nLSU	mtSSU	*rpb*2	*tef*1-*α*	
*Abortiporus biennis*	Cui 16986	ON417150	ON417198	ON417065	ON424751	ON424822	Liu et al. (2023a)
*Amylocorticium cebennense*	HHB-2808	GU187505	GU187561	GU187439	GU187770	GU187675	Wang et al. (2023)
*Anomoloma myceliosum*	MJL-4413	GU187500	GU187559		GU187766	GU187677	Wang et al. (2023)
*Antella americana*	KHL 11949	JN710509	JN710509	JN710656		JN710711	Miettinen et al. (2012)
*Antella chinensis*	Dai 8874	JX110843	KC485541				Schoch et al. (2014)
*Antella chinensis*	Dai 9019	NR_120162	NG_057020				Schoch et al. (2014)
*Antrodiella faginea*	3165	JN710514	JN710514	JN710658		JN710712	Miettinen et al. (2012)
*Antrodiella onychoides*	X 155	NR_120165	JN710517	JN710660			Schoch et al. (2014)
*Antrodiella pallescens*	X1080	NR_120166	JN710518	JN710661			Schoch et al. (2014)
*Antrodiella romellii*	X 154	NR_120167	JN710520				Schoch et al. (2014)
*Antrodiella semisupina*	X 242	JN710521	JN710521				Miettinen et al. (2012)
*Antrodiella stipitata*	FD 136	KP135314	KP135197				Liu et al. (2023b)
*Antrodiella stipitata*	Yuan 5640	NR_120169	KC485544				Schoch et al. (2014)
*Aroramyces gelatinosporus*	H4010		DQ218524		DQ218941	DQ219118	Wang et al. (2023)
*Asterophora lycoperdoides*	CBS 170.86	AF357037	AF223190		DQ367431	DQ367424	Wang et al. (2023)
*Athelia arachnoidea*	CBS 418.72	GU187504	GU187557		GU187769	GU187672	Wang et al. (2023)
*Atraporiella neotropica*	X1021	HQ659221	HQ659221				Miettinen and Rajchenberg (2012)
*Atraporiella yunnanensis*	CLZhao 604	MF962482	MF962485	MZ958849	OK000939	OK000966	Wu et al. (2017)
*Atraporiella yunnanensis*	CLZhao 605	NR_120172	NG_058606	MZ958850	OK000940		Wu et al. (2017)
*Auricularia heimuer*	Xiaoheimao	LT716074	KY418890		KY419035	KY419083	Wang et al. (2023)
*Boletopsis leucomelaena*	PBM2678	DQ484064	DQ154112		GU187820	GU187763	Wang et al. (2023)
*Bondarzewia occidentalis*	AFTOL-ID 452	DQ200923	DQ234539		AY218474	DQ059044	Matheny et al. (2007)
*Bondarzewia occidentalis*	HHB-14803	KM243329	KM243332	KX066176	KX066163	KX066142	Chen et al. (2016)
*Butyrea japonica*	10202	JN710556	JN710556	JN710680		JN710718	Miettinen et al. (2012)
*Butyrea luteoalba*	5403	JN710558	JN710558	JN710682		JN710719	Cao et al. (2021)
*Butyrea luteoalba*	FP-105786	KP135320	KP135226		KP134963		Miettinen et al. (2012)
*Byssomerulius corium*	FP-102382	KP135007	KP135230			KP134921	Floudas and Hibbett (2015)
*Byssomerulius corium*	Wu 1708-327	LC427007	LC427031				Chen et al. (2020)
*Calocera cornea*	AFTOL 438	AY789083	AY701526		AY536286	AY881019	Wang et al. (2023)
*Ceriporia arbuscula*	GC 1708338*	LC427008	LC427040				Chen et al. (2020)
*Ceriporia aurantiocarnescens*	JV 0105/10	KX236482	KX236482				Wang et al. (2023)
*Ceriporia crassa*	Dai 22034*	OQ476823	OQ476769		OQ559579		Wang et al. (2023)
*Ceriporia excelsa*	CBS:344.63	MH858306	MH869917				Vu et al. (2019)
*Ceriporia hinnulea*	Cui 11291*	OQ476826	OQ476772		OQ559583		Wang et al. (2023)
*Ceriporia punctata*	Dai 15899*	OQ476839	OQ476784	OQ509551		OQ559588	Wang et al. (2023)
*Ceriporia viridans*	Miettinen 11701	KX752600	KX752600				Miettinen and Ryvarden (2016)
*Ceriporiopsis aneirina*	HHB-15629-Sp	KP135023	KP135207				Larsson et al. (2025)
*Cerrena unicolor*	He 6082	OM100740	OM083972	ON417068	ON424756	ON424825	Liu et al. (2022b)
*Cerrena zonata*	Cui 18502	ON417154	ON417204	ON417070	ON424758	ON424827	Liu et al. (2022b)
*Chondrogaster pachysporus*	OSC49298		DQ218538		DQ218958	DQ219136	Wang et al. (2023)
*Citripora bannaensis*	CLZhao 595	MG231568	MG748854				Wu et al. (2018)
*Citripora bannaensis*	CLZhao 596	MG231572	MG748855				Wu et al. (2018)
*Clavariadelphus truncatus*	OSC67280		AY574649		DQ219064	DQ219240	Wang et al. (2023)
*Climacocystis borealis*	Dai 4014	KJ566627	KJ566637			KJ566644	Liu et al. (2023a)
*Climacocystis montana*	Cui 17122	ON682359	ON680811		ON688485	ON688505	Liu et al. (2023a)
*Coniophora arida*	FP104367	GU187510	GU187573		GU187775	GU187684	Wang et al. (2023)
*Cytidiella albida*	GB-1833	KY948748	KY948889				Justo et al. (2017)
*Cytidiella albomarginata*	WEI 18-474	MZ636948	MZ637110		OK136070		Chen et al. (2021)
*Cytidiella albomarginata*	He 5575	MZ422526	MZ422497				Li et al. (2022)
*Cytidiella albomellea*	He 3089	MZ422525	MZ422496				Li et al. (2022)
*Cytidiella nitidula*	He 5126	MZ422523	MZ422494				Li et al. (2022)
*Cytidiella nitidula*	He 5135	MZ422524	MZ422495				Li et al. (2022)
*Dacryopinax spathularia*	AFTOL 454	AY854070	AY701525		AY857981	AY881020	Wang et al. (2023)
*Efibula clarkii*	FD-228	KP135019					Floudas and Hibbett (2015)
*Efibula cremea*	ClZhao 19298	PV759509	PV857760				Li et al. (2025a)
*Efibula daweishanensis*	CLZhao 18946	OR094488					Dong et al. (2024)
*Efibula daweishanensis*	CLZhao 19002	OR094489	OR449958				Dong et al. (2024)
*Efibula daweishanensis*	CLZhao 25072	OR094490	OR449959	OR469100	OR733284	OR541913	Dong et al. (2024)
*Efibula glossophora*	CLZhao 22744 *	PV470540	PV474185		PV759036	PV759050	Gu et al. (2025)
*Efibula gracilis*	FD-455	KP135027	MZ637116		OK136077	MZ913679	Floudas and Hibbett (2015)
*Efibula gracilis*	FP-102052	KP135028					Floudas and Hibbett (2015)
*Efibula grandinosa*	BJFC 033256	NR182914	NG149002				Larsson et al. (2025)
*Efibula grandinosa*	He 6312s*	MZ422509	MZ422480				Li et al. (2022)
*Efibula hainanensis*	BJFC 030880	NR_182875	NG148987				Li et al. (2022)
*Efibula hainanensis*	He 6004*	MW580949	MW580939				Li et al. (2022)
*Efibula intertexta*	Wu 1707-93	MZ636954	MZ637117		OK136085		Chen et al. (2021)
*Efibula intertexta*	Wu 1707-96	MZ636953	MZ637118		OK136086		Chen et al. (2021)
*Efibula matsuensis*	Wu 1011-18	MZ636956	MZ637119		OK136078	MZ913680	Chen et al. (2021)
*Efibula matsuensis*	Wu 1011-19	MZ636957	MZ637120				Larsson et al. (2025)
*Efibula murina*	CLZhao 30689*	PP780187	PP785353	PV774704			Present study
*Efibula murina*	CLZhao 35686	PQ404901	PV771613	PV774705			Present study
*Efibula murina*	CLZhao 35695	PQ404902	PV771614	PV774706			Present study
*Efibula murina*	CLZhao 35707	PQ404903	PV771615	PV774707			Present study
*Efibula punctata*	CLZhao 30011	PV470544	PV474189				Gu et al. (2025)
*Efibula punctata*	CLZhao 30054	PV470545	PV474190		PV759041	PV759055	Gu et al. (2025)
*Efibula shenghuae*	He 3384*	MZ422508	MZ422479				Li et al. (2022)
*Efibula tropica*	Chen 3596	MZ636966	MZ637128				Chen et al. (2021)
*Efibula tropica*	WEI 18-149	MZ636967	MZ637129		OK136079	MZ913681	Chen et al. (2021)
*Efibula tuberculata*	Wu 0711-148	MZ636969	MZ637131			MZ913671	Larsson et al. (2025)
*Efibula tuberculata*	Wu 1005-55	MZ636970	MZ637132		OK136074	MZ913672	Chen et al. (2021)
*Efibula turgida*	Wu 0910-86	MZ636972	MZ637134				Chen et al. (2021)
*Efibula turgida*	Wu 0910-99	MZ636973	MZ637135				Chen et al. (2021)
*Efibula yaoshanensis*	CLZhao 20575*	PP780185	PP785351	PP785355		PQ723765	Present study
*Efibula yunnanensis*	He 4653	MW580948	MW580938				Li et al. (2022)
*Efibula yunnanensis*	He 6970	MZ422505	MZ422476				Li et al. (2022)
*Efibula yunnanensis*	Wu 880515-1	MZ636977	GQ470672		MZ748420	MZ913682	Liu et al. (2023b)
*Efibula zhaotongensis*	CLZhao 20744*	PQ404904	PQ404895	PQ404891			Present study
*Efibula zhaotongensis*	CLZhao 38003	PQ404905	PV771616	PV774708			Present study
*Etheirodon fimbriatum*	HR 98811	MT849300				MT833938	Westphalen et al. (2021)
*Etheirodon fimbriatum*	KHL 11905	JN710530	JN710530	JN710667			Miettinen et al. (2012)
*Etheirodon purpureum*	MCW 642/18	MT849301	MT849301			MT833939	Westphalen et al. (2021)
*Exidia* sp.	PBM2527	DQ241774	AY700191			DQ408144	Wang et al. (2023)
*Flaviporus brownii*	MCW 362/12	KY175008	KY175008			KY175022	Westphalen et al. (2021)
*Flaviporus brownii*	X 462	JN710538	JN710538	JN710670		JN710715	Miettinen et al. (2012)
*Flaviporus liebmannii*	AS1567	KY969753	KY969740				Unpublished
*Flaviporus liebmannii*	X 666	JN710540	JN710540				Miettinen et al. (2012)
*Flaviporus tenuis*	MCW 356/12	KY175002	KY175002				Westphalen et al. (2018)
*Flaviporus tenuis*	MCW 442/13	KY175001	KY175001				Westphalen et al. (2018)
*Fomitiporia mediterranea*	AFTOL688	AY854080	AY684157		AY803748	AY885149	Wang et al. (2023)
*Frantisekia fissiliformis*	CBS 435.72	MH860521	MH872232				Vu et al. (2019)
*Frantisekia mentschulensis*	BRNM 710170	FJ496670	FJ496728	FJ496748			Tomšovský et al. (2010)
*Frantisekia ussurii*	Dai 8249	KC485526					Yuan (2014)
*Frantisekia ussurii*	Wei 3081	KC485527	KC485545				Yuan (2014)
*Geasteroides taylorii*	OSC59760		DQ218520		DQ219060	DQ219235	Wang et al. (2023)
*Gloeoporellus merulinus*	Cui 16629	ON682364	ON680816	OQ534089	ON688492	ON688512	Liu et al. (2023b)
*Gloeoporellus merulinus*	Cui 16650	ON682365	ON680817	OQ534090	ON688493	ON688513	Liu et al. (2023b)
*Gloeoporus hainanensis*	Dai 15268*	KU360401	KU360411	OQ509569		OQ559601	Wang et al. (2023)
*Gloeoporus orientalis*	Cui 11339	OQ476855	OR088496	OQ509571		OQ559603	Wang et al. (2023)
*Gomphidius roseus*	MB 95-038	DQ534570	DQ534669		GU187818	GU187702	Wang et al. (2023)
*Gymnopilus picreus*	ZRL2015011	LT716066	KY418882		KY419027	KY419077	Wang et al. (2023)
*Heterobasidion annosum*	Korhonen 06129/6	KJ583211	KJ583225	KJ651577	KF006499	KX252741	Chen et al. (2016)
*Hypochnicium bombycinum*	Otto Miettinen 9441 (H)	KY415959	KY415959				Maekawa et al. (2023)
*Hypochnicium karstenii*	NH 10924	DQ677510	DQ677510				Larsson (2007)
*Irpex alboflavescens*	He 3933*	MZ422503	MZ422474				Li et al. (2022)
*Irpex flavus*	Wu 0705-1	MZ636988	MZ637149		OK136087	MZ913683	Liu et al. (2022b)
*Irpex hydnoides*	KUC20121109-01	KJ668510	KJ668362				Jang et al. (2016)
*Irpex jinshaensis*	Dai 22402	MZ787973	MZ787965				Tian et al. (2022)
*Irpex laceratus*	Dai 16433	OQ476861	OQ476803	OQ509576		OQ559608	Wang et al. (2023)
*Irpex laceratus*	Dai 21940	OQ476862	OQ476804	OQ509577		OQ559609	Wang et al. (2023)
*Irpex lacteus*	Dai 11230	OQ476863	OQ476805	OQ509578		OQ559610	Wang et al. (2023)
*Irpex latemarginatus*	Marcin Piatek 4.IX.1997	KX752592	KX752592				Miettinen et al. (2016)
*Irpex lenis*	Wu 1608-22	MZ636992	MZ637153				Chen et al. (2021)
*Irpex rosea*	CLZhao 18491*	MW377575	MW377578				Wang and Zhao (2021)
*Irpex rosettiformis*	LR40855	JN649347	JN649347				Sjökvist et al. (2012)
*Junghuhnia crustacea*	X1127	JN710554	JN710554	JN710678			Miettinen et al. (2012)
*Junghuhnia crustacea*	X262	JN710553	JN710553				Miettinen et al. (2012)
*Junghuhnia pseudocrustacea*	Yuan 6160	MF139551					Yuan et al. (2019)
*Junghuhnia pseudocrustacea*	Zhou 283	MF139552					Yuan et al. (2019)
*Kavinia alboviridis*	0102140		AY574692		DQ219073	DQ219250	Wang et al. (2023)
*Leptosporomyces raunkiaerii*	HHB-7628	GU187528	GU187588		GU187791		Wang et al. (2023)
*Loweomyces fractipes*	X1253	JN710569	JN710569	JN710689			Miettinen et al. (2012)
*Loweomyces spissus*	MCW 488/14	KX378869	KX378869				Westphalen et al. (2016)
*Loweomyces tomentosus*	MCW 366/12	KX378870	KX378870				Westphalen et al. (2016)
*Meripilus giganteus*	FP 135344	KP135307	KP135228				Floudas and Hibbett (2015)
*Meripilus longicystidius*	Cui 16725	ON417178	ON417228	ON417042	ON424796	ON424857	Liu et al. (2023a)
*Metuloidea cinnamomea*	X 1228	KU926963					Miettinen and Ryvarden (2016)
*Metuloidea fragrans*	BRNM 826045	MW565825					Tomšovský et al. (2021)
*Metuloidea murashkinsky*	X449	JN710588	JN710588				Westphalen et al. (2019)
*Metuloidea murashkinskyi*	CLZhao 9455	MT247001					Unpublished
*Metuloidea reniforme*	MCW 523/17	MT849302	MT849302				Westphalen et al. (2021)
*Metuloidea reniforme*	MCW 542/17	MT849303	MT849303			MT833940	Westphalen et al. (2021)
*Metuloidea rhinocephala*	X460	JN710562	JN710562				Westphalen et al. (2019)
*Mycorrhaphium adustum*	KHL 12255	JN710573	JN710573	JN710692		JN710727	Miettinen et al. (2012)
*Mycorrhaphium subadustum*	Dai 10173	KC485537	KC485554				Yuan (2014)
*Mycorrhaphium subadustum*	Yuan 12976	MW491378	MW488040				Cao et al. (2021)
*Neohypochnicium murinum*	CLZhao 19017	OQ788985	OQ789005	OR469109		OR541917	Dong et al. (2024)
*Neohypochnicium perlongicystidiosum*	TUMH:40397	LC663679	LC663690				Maekawa et al. (2023)
*Neurospora crassa*	OR74A	HQ271348	AF286411		AF107789	XM959775	Wang et al. (2023)
*Nigroporus stipitatus*	X546	JN710574	JN710574				Miettinen et al. (2012)
*Nigroporus vinosus*	8182	JN710575	JN710575	JN710693		JN710728	Miettinen et al. (2012)
*Nigroporus vinosus*	Yuan12916	MT681923	MT675108		MT793116	MT793113	Cao et al. (2021)
*Odentinium aurantium*	CLZhao 20737*	PQ404911	PQ404898	PQ404893			Present study
*Odentinium aurantium*	CLZhao 38004	PV771629					Present study
*Odentinium cremeum*	CLZhao 20573*	PQ404906	PQ404897	PQ404892			Present study
*Odentinium cremeum*	CLZhao 20648	PQ404907					Present study
*Odentinium cremeum*	CLZhao 26660	PQ404908	PQ404896				Present study
*Odentinium cremeum*	CLZhao 31635	PQ404909	PV771617	PV774709			Present study
*Odentinium cremeum*	CLZhao 32563	PQ404910					Present study
*Panus conchatus*	Dai 23421	ON417176	ON417226	ON417088	ON424794	ON424855	Liu et al. (2022b)
*Panus fragilis*	HHB 11042	KP135328	KP135233				Floudas and Hibbett (2015)
*Phanericium americanum*	FP-102165	KP135016	KP135256				Larsson et al. (2025)
*Phanericium americanum*	HHB-10209-Sp	KP135014					Larsson et al. (2025)
*Phanericium bambusacearum*	CLZhao 20795*	PP780186	PP785352	PP785356		PQ720668	Present study
*Phanericium rodriguezarmasiae*	MA-Fungi 86626	KF483015	KF528106				Larsson et al. (2025)
*Phanericium subglobisporum*	Chen 1716	MZ636962	MZ637124		OK136075	MZ913673	Larsson et al. (2025)
*Phanericium subglobisporum*	He 3983	MW580944	MW580934				Larsson et al. (2025)
*Phanericium taiwanensis*	He 4582a	MZ422507	MZ422478				Larsson et al. (2025)
*Phanericium tuberculatum*	FCUG305	MZ636959	GQ470669				Larsson et al. (2025)
*Phanerochaetella angustocystidiata*	He 2965	MZ422515	MZ422486				Li et al. (2022)
*Phanerochaetella formosana*	He 3391	MZ422520	MZ422491				Larsson et al. (2025)
*Phanerochaetella formosana*	He 3962	MZ422522	MZ422493				Li et al. (2022)
*Phellinus hartigii*	Dai 11766	KT203287	KT203308	KT203329	KJ651721		Wang et al. (2023)
*Podoscypha venustula*	Cui 16923	ON417181	ON417231	ON417089	ON424799	ON424860	Liu et al. (2022b)
*Rhomboidia wuliangshanensis*	CLZhao 4406	MK860715	MK860710				Xu et al. (2020)
*Rhomboidia wuliangshanensis*	CLZhao 4411	MK860716	MK860711				Xu et al. (2020)
*Sarcoporia polyspora*	Cui 16977	MW377326	MW377403		MW337079	MW337146	Liu et al. (2022b)
*Sarcoporia polyspora*	Cui 16995	OM039299	OM039199		ON424811	OM037817	Liu et al. (2022b)
*Schenella pityophilus*	OSC59743		DQ218519	DQ218694	DQ219057	DQ219232	Wang et al. (2023)
*Sebacina* sp.	AFTOL 1517	DQ911617	DQ521412				Wang et al. (2023)
*Skeletocutis coprosmae*	Cui 16623	ON417193	ON417245	ON417100	ON424813	ON424879	Liu et al. (2022b)
*Skeletocutis nivea*	Cui 16752	ON682369	ON680821		ON688497	ON688517	Liu et al. (2023a)
*Steccherinum bourdotii*	CLZhao 924	MG231819	MZ713807	MZ958869		OK000971	Unpublished
*Steccherinum bourdotii*	HR102002	MT849310				MT833946	Westphalen et al. (2021)
*Steccherinum hirsutum*	CLZhao 4222	MW290040	MW290054	MZ958871	OK000954	OK000973	Dong et al. (2022)
*Steccherinum hirsutum*	CLZhao 4523	MW290041	MW290055	MZ958872	OK000955		Dong et al. (2022)
*Steccherinum larssonii*	MCW 593/17	MT849306	MT849306		MT833956	MT833941	Liu et al. (2023b)
*Steccherinum meridionale*	Cui 16691	ON417195	ON417247		ON424743	ON424882	Liu et al. (2023b)
*Steccherinum ochraceum*	2060	JN710589	JN710589				Miettinen et al. (2012)
*Steccherinum ochraceum*	KHL 11902	JN710590	JN710590	JN710700	JN710738	JN710730	Miettinen et al. (2012)
*Steccherinum tenue*	KHL 12316	JN710598	JN710598	JN710705	JN710739	JN710733	Miettinen et al. (2012)
*Steccherinum yunnanense*	CLZhao 1445	MW290042	MW290056	MZ958889		OK000984	Dong et al. (2022)
*Stereum hirsutum*	AFTOL-ID 492	AY854063			AY218520	AY885159	Liu et al. (2023a)
*Thelephora ganbajun*	ZRL20151295	LT716082	KY418908		KY419043	KY419093	Wang et al. (2023)
*Trametopsis aborigena*	Robledo 1236	KY655336	KY655338				Lopes et al. (2017)
*Trametopsis cervina*	TJV-93-216T	JN165020	JN164796		JN164877	JN164882	Justo and Hibbett (2011)
*Tremellodendron pallidum*	AFTOL 699	DQ411526	AY745701		DQ408132	DQ029196	Wang et al. (2023)
*Trullella conifericola*	Yuan 12657	MT269761	MT259327			MT793110	Cao et al. (2021)
*Trullella dentipora*	X200	JN710512	JN710512				Miettinen et al. (2012)
*Trullella duracina*	Dai 20474	OL437266	OL434415				Unpublished
*Trullella duracina*	MCW 410/12	MH475309	MH475309				Westphalen et al. (2019)
*Xanthoporus syringae*	1488	JN710607	JN710607				Miettinen et al. (2012)
*Xanthoporus syringae*	X339	JN710606	JN710606				Miettinen et al. (2012)
*Xenasmatella gossypina*	LWZ 2020081825b	OQ738196	OQ674442	OQ758235	OQ683410		Liu et al. (2023c)
*Xenasmatella hjortstami*	LWZ 2020081929a	OQ738199	OQ674443	OQ758236		OQ683409	Liu et al. (2023c)

### ﻿Phylogenetic analyses

Taxa for phylogenetic analyses were sampled from *Polyporales* (Cui et al. 2019; Li et al. 2022; Liu et al. 2023a). For a preliminary assessment of taxonomic affiliation, all newly obtained sequences were tested using NCBI BLAST. DNA sequences were aligned in MAFFT version 7 using the G-INS-i strategy (Katoh et al. 2019). The alignment was manually adjusted using AliView version 1.27 (Larsson 2014). The datasets were initially aligned separately and later combined using Mesquite version 3.51.

For phylogenetic analyses, maximum likelihood (ML), maximum parsimony (MP), and Bayesian inference (BI) were used. The approaches to the phylogenetic analysis process followed Dong et al. (2024). MP analysis was performed in PAUP* version 4.0b10 (Swofford 2002). All characters were equally weighted, and gaps were treated as missing data. Trees were inferred using the heuristic search option with tree bisection–reconnection branch swapping and 1000 random sequence additions. The maximum number of trees was set to 5000, branches of zero length were collapsed, and all most parsimonious trees were saved. Clade robustness was assessed using bootstrap analysis with 1000 replicates (Felsenstein 1985). Descriptive tree statistics, including tree length (TL), consistency index (CI), retention index (RI), rescaled consistency index (RC), and homoplasy index (HI), were calculated. BI was performed using MrBayes version 3.1.2 (Zhao et al. 2023a). ML analyses were conducted using RAxML-HPC BlackBox version 8.2.12 through the CIPRES Science Gateway (Miller et al. 2012). Datasets were analyzed in MrBayes version 3.2.7a (Ronquist et al. 2012), implementing the best-fit model of nucleotide evolution for each partition as inferred from jModelTest version 2 (Ronquist and Huelsenbeck 2003; Nylander 2004; Darriba et al. 2012). Four Markov chains were run for two independent runs from random starting trees until the average standard deviation of split frequencies dropped below 0.01. The first quarter of generations was discarded as burn-in, and a majority-rule consensus tree was computed from the remaining trees. Branches were considered significantly supported if they received ML bootstrap support (BS) ≥ 70%, MP bootstrap support (BT) ≥ 50%, or Bayesian posterior probability (BPP) ≥ 0.95.

In the phylogenetic analyses, a five-gene dataset (ITS+nLSU+mtSSU+*rpb*2+*tef*1-α) was used to determine the phylogenetic position of the new genus within *Steccherinaceae*, following Miettinen et al. (2012), Cao et al. (2021), and Westphalen et al. (2021). Additionally, due to the high sequence coverage of ITS and nLSU in *Phanericium* and *Efibula* species, a two-gene dataset (ITS+nLSU) was used to determine the phylogenetic positions of the new *Phanericium* and *Efibula* species within *Irpicaceae*, based on Li et al. (2022) and Larsson et al. (2025). Furthermore, a combined dataset of ITS, nLSU, mtSSU, *rpb*2, and *tef*1-α sequences was used to estimate divergence times of families within *Polyporales* using molecular clock analyses, following Liu et al. (2023) and Wang et al. (2024).

### ﻿Divergence time estimation

Three fossil calibrations, *Archaeomarasmius leggetti* Hibbett, D. Grimaldi and Donoghue, *Quatsinoporites cranhamii* S.Y. Sm., Currah and Stockey, and *Paleopyrenomycites devonicus* Taylor, Hass, Kerp, M. Krings and Hanlin, were used for divergence time estimation. *Archaeomarasmius leggetti* was used as the representative of the minimum age of *Agaricales* at 90 Mya (Hibbett et al. 1997); *Q. cranhamii* was used as the representative of the minimum age of *Hymenochaetaceae* at 125 Mya (Dong et al. 2024); and *P. devonicus* was used as the representative of the minimum age between Basidiomycota and *Ascomycota* at 400 Mya (Taylor et al. 2005; Berbee and Taylor 2010). Divergence time was estimated using the BEAST v2.6.5 software package (Bouckaert et al. 2014) with ITS, nLSU, mtSSU, *rpb*2, and *tef*1-α sequences representing the main lineages in *Polyporales*. According to these calibration points, the offset age with a gamma distribution prior (scale = 20, shape = 1) for *Agaricales* was set to 90 Mya, and that for *Hymenochaetaceae* was set to 125 Mya. Analyses were run for 30 million generations. The log file was analyzed in Tracer v1.6 to confirm that the estimated effective sample size (ESS) was ≥ 200^3^. The first 10% of sampled trees, sampled every 1000^th^ generation, were removed as burn-in. The resulting log file was further checked for chain convergence using Tracer v1.5.

## ﻿Results

### ﻿Phylogenetic analyses

**Figure 1.**
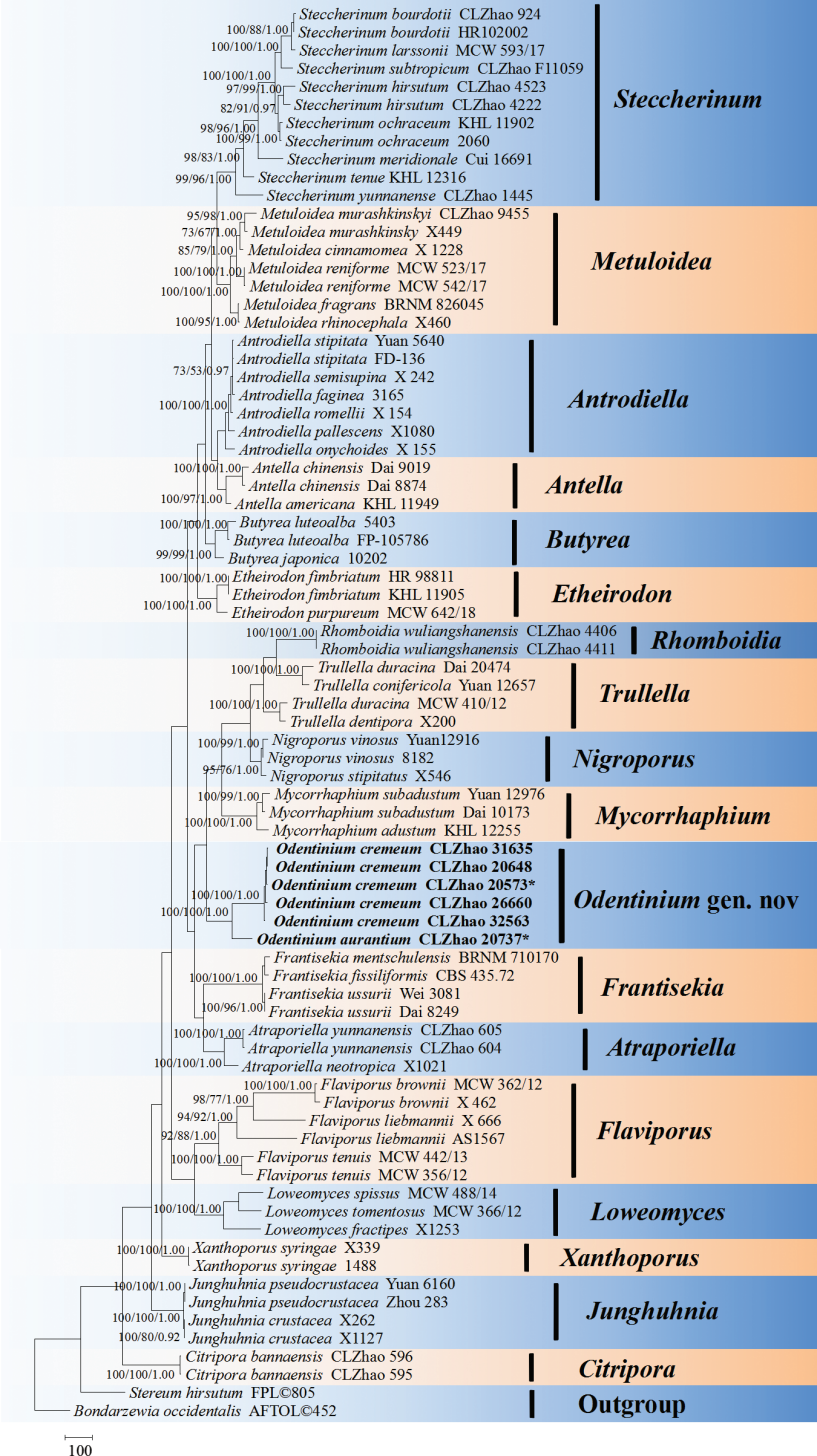
Maximum parsimony strict consensus tree illustrating the phylogeny of the new genus *Odentinium* and related species within *Steccherinaceae* based on ITS, nLSU, mtSSU, *rpb*2, and *tef*1-α sequences. Branches are labeled with maximum likelihood bootstrap values higher than 70%, parsimony bootstrap values higher than 50%, and Bayesian posterior probabilities higher than 0.95, respectively. The new species are shown in bold.

The aligned dataset encompassed 78 specimens representing 52 taxa, including two new species, and the taxa *Stereum hirsutum* and *Bondarzewia occidentalis* Jia J. Chen, B.K. Cui & Y.C. Dai were retrieved from GenBank as outgroups for analysis using the concatenated ITS, nLSU, mtSSU, *rpb*2, and *tef*1-α sequence dataset (Fig. 1), following a previous study (Cao et al. 2021). Four Markov chains were run for two runs from random starting trees, each for 4 million generations. The dataset had an aligned length of 6731 characters, of which 4713 characters were constant, 663 were variable and parsimony uninformative, and 1355 were parsimony informative. Maximum parsimony analysis yielded one equally parsimonious tree (TL = 6607, CI = 0.4301, HI = 0.5699, RI = 0.6625, RC = 0.2850). The best-fit model was GTR+I+G for the ITS, nLSU, mtSSU, *rpb*2, and *tef*1-α datasets, which was estimated and applied in the Bayesian analysis. Both Bayesian inference and ML analyses resulted in a topology similar to that obtained from MP analysis, with an average standard deviation of split frequencies of 0.007615 (BI), and the effective sample size (ESS) across the two runs was double the average ESS (avg ESS = 825.92).

The phylogram depicts the overall topology of the family *Steccherinaceae* (Fig. 1), based on the ITS, nLSU, mtSSU, *rpb*2, and *tef*1-α dataset. The species represented belong to 17 previously known genera, viz., *Antella* Miettinen, *Antrodiella* Ryvarden & I. Johans., *Atraporiella* Ryvarden, *Butyrea* Miettinen, *Citripora* Miettinen, *Etheirodon* Banker, *Flaviporus* Murrill, *Frantisekia* Spirin & Zmitr., *Junghuhnia* Corda, *Loweomyces* (Kotl. & Pouzar) Jülich, *Metuloidea* G. Cunn., *Mycorrhaphium* Maas Geest., *Nigroporus* Murrill, *Rhomboidia* C.L. Zhao, *Steccherinum* Gray, *Trullella* Zmitr., and *Xanthoporus* Audet, together with the new genus *Odentinium*. The phylogenetic tree (Fig. 1) showed that six specimens of the new genus *Odentinium* formed a distinct lineage with strong support within *Steccherinaceae*, and that the two new species, *Odentinium cremeum* Y.L. Deng & C.L. Zhao and *O. aurantium* Y.L. Deng & C.L. Zhao, formed a single clade with strong support.

**Figure 2.**
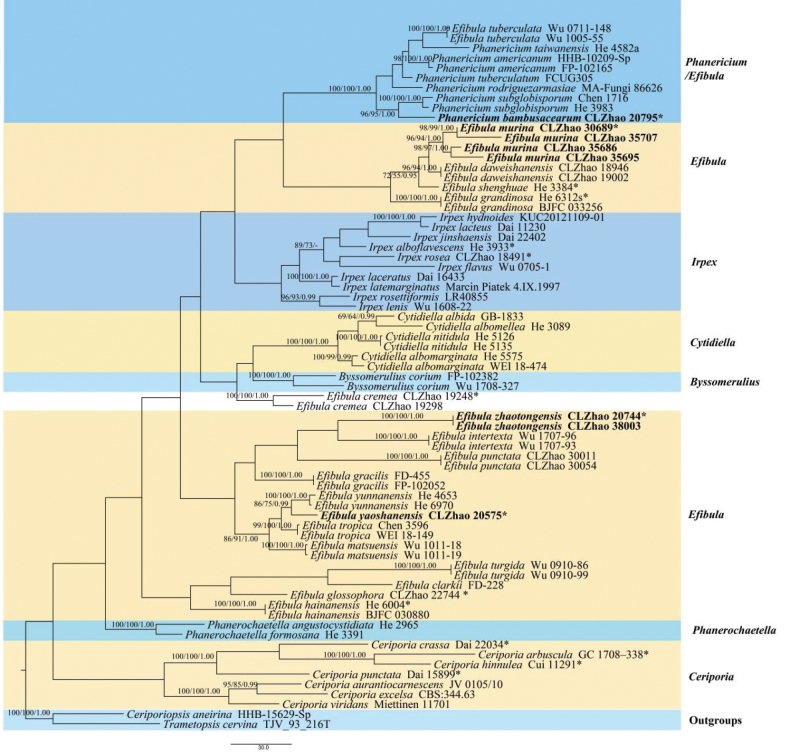
Maximum parsimony strict consensus tree illustrating the phylogeny of *Irpicaceae* based on ITS+nLSU sequences. Branches are labeled with maximum likelihood bootstrap values higher than 70%, parsimony bootstrap values higher than 50%, and Bayesian posterior probabilities higher than 0.95, respectively. The new species are shown in bold.

The phylogram of *Irpicaceae* based on the combined ITS+nLSU dataset is shown in Fig. 2. The aligned dataset encompassed 71 specimens representing 50 taxa, including four new species, *E. murina*, *E. yaoshanensis*, *E. zhaotongensis*, and *Phanericium bambusacearum*. The outgroups *Bondarzewia occidentalis* and *Stereum hirsutum* were retrieved from GenBank, following a previous study (Larsson et al. 2025). The trees inferred from the three analyses showed identical topologies. Four Markov chains were run for two runs from random starting trees, each for 1.725 million generations. The dataset had an aligned length of 2133 characters, of which 1459 characters were constant, 205 were variable and parsimony uninformative, and 469 were parsimony informative. Maximum parsimony analysis yielded two equally parsimonious trees (TL = 2548, CI = 0.3850, HI = 0.6150, RI = 0.6525, RC = 0.2512). The best-fit model was GTR+I+G for the ITS+nLSU dataset, which was estimated and applied in the Bayesian analysis. Both Bayesian inference and ML analyses resulted in a topology similar to that obtained from MP analysis, with an average standard deviation of split frequencies of 0.009884 (BI), and the effective sample size (ESS) across the two runs was double the average ESS (avg ESS = 2017). The phylogram depicts the overall topology of the family *Irpicaceae* (Fig. 2), and species from seven previously known genera, viz., *Byssomerulius* Parmasto, *Ceriporia* Donk, *Cytidiella* Pouzar, *Efibula*, *Irpex*, *Phanericium*, and *Phanerochaetella* C.C. Chen & Sheng H. Wu, were recovered. In the molecular phylogenetic analyses, *Efibula* formed two clades, and the three new species, *E. murina*, *E. yaoshanensis*, and *E. zhaotongensis*, were grouped within *Efibula* with strong support. Moreover, the new species *Phanericium bambusacearum* formed a well-supported lineage within *Phanericium*, and *Phanericium* was clustered with *Efibula*, which agrees with a previous study by Larsson et al. (2025).

**Figure 3.**
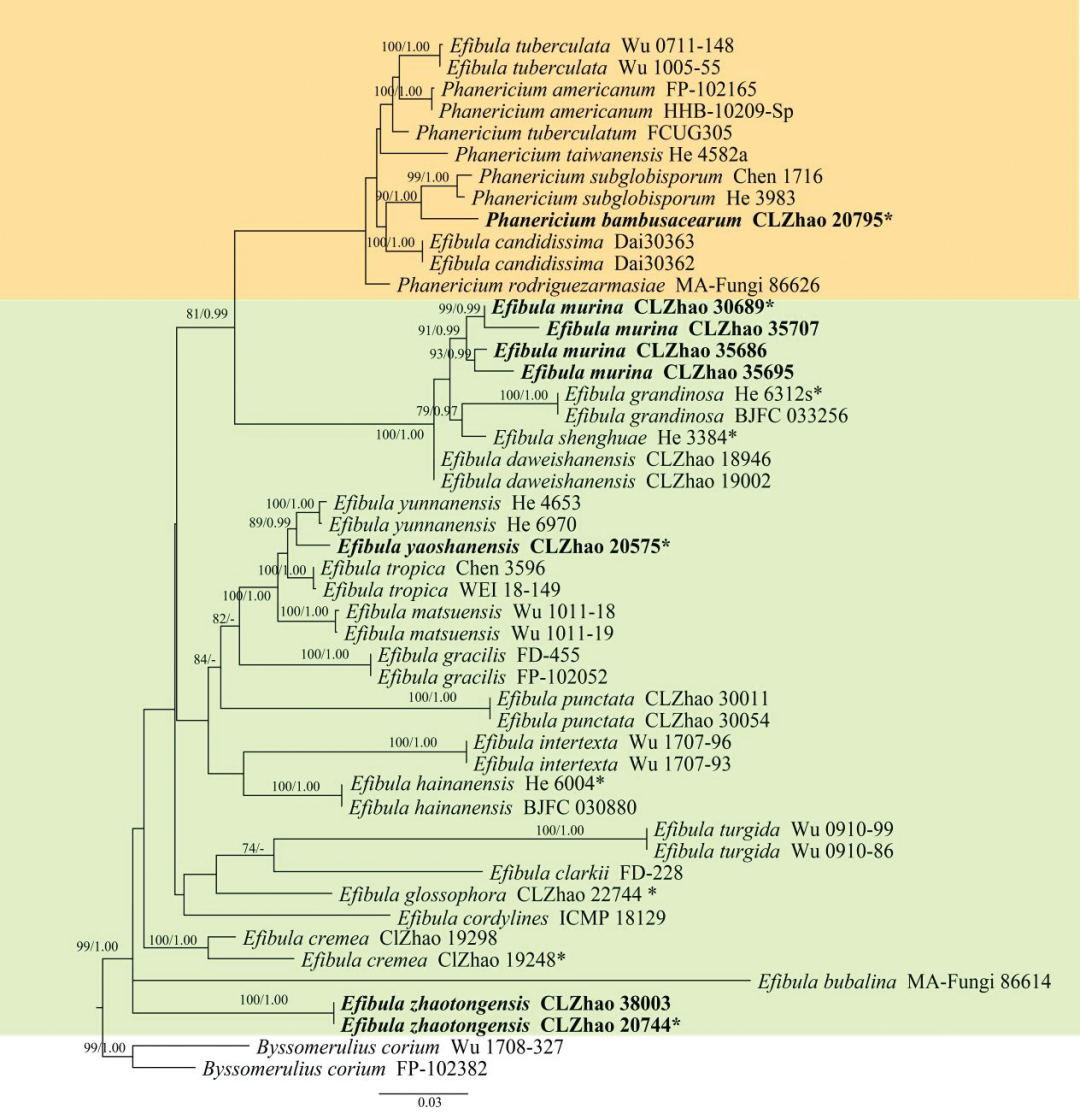
Maximum parsimony strict consensus tree illustrating the phylogeny of *Efibula* and related species based on ITS+nLSU sequences. Branches are labeled with maximum likelihood bootstrap values higher than 70% and Bayesian posterior probabilities higher than 0.95, respectively. The new species are shown in bold.

The aligned dataset encompassed 48 specimens representing 28 taxa, including four new species, and the outgroup species *Byssomerulius corium* (Pers.) Parmasto was retrieved from GenBank (Fig. 3), following a previous study (Chen et al. 2021). Four Markov chains were run for two runs from random starting trees, each for 0.64 million generations. The best RAxML tree, with a final likelihood value of −9917.121433, is presented. The evolutionary model TPM2uf+I+G was applied for all genes. The matrix contained 625 distinct alignment patterns, with 23.33% undetermined characters or gaps. Estimated base frequencies were as follows: A = 0.264968, C = 0.200070, G = 0.263838, and T = 0.271124; substitution rates were AC = 1.451831, AG = 3.016263, AT = 1.592906, CG = 0.822064, CT = 6.351639, and GT = 1.000000; the gamma distribution shape parameter (α) was 0.201417. Bayesian inference and ML analyses resulted in a topology similar to that obtained from MP analysis, with an average standard deviation of split frequencies of 0.009289 (BI), and the effective sample size (ESS) across the two runs was double the average ESS (avg ESS = 1302). The phylogenetic tree (Fig. 3) inferred from the ITS+nLSU sequences revealed that the new species *Efibula murina* grouped with two taxa, *E. grandinosa* Yue Li & S.H. He and *E. shenghuae* Yue Li & S.H. He. The new taxon *E. yaoshanensis* was recovered as sister to *E. yunnanensis* C.L. Zhao. The new species *E. zhaotongensis* formed a clade and was closely related to *E. bubalina* (Burds.) Zmitr. & Spirin. Moreover, the species *Phanericium bambusacearum* was grouped within *Phanericium* and was sister to *P. subglobisporum* (C.C. Chen & Sheng H. Wu) K.H. Larss. & Spirin.

### ﻿Divergence time estimation

**Figure 4.**
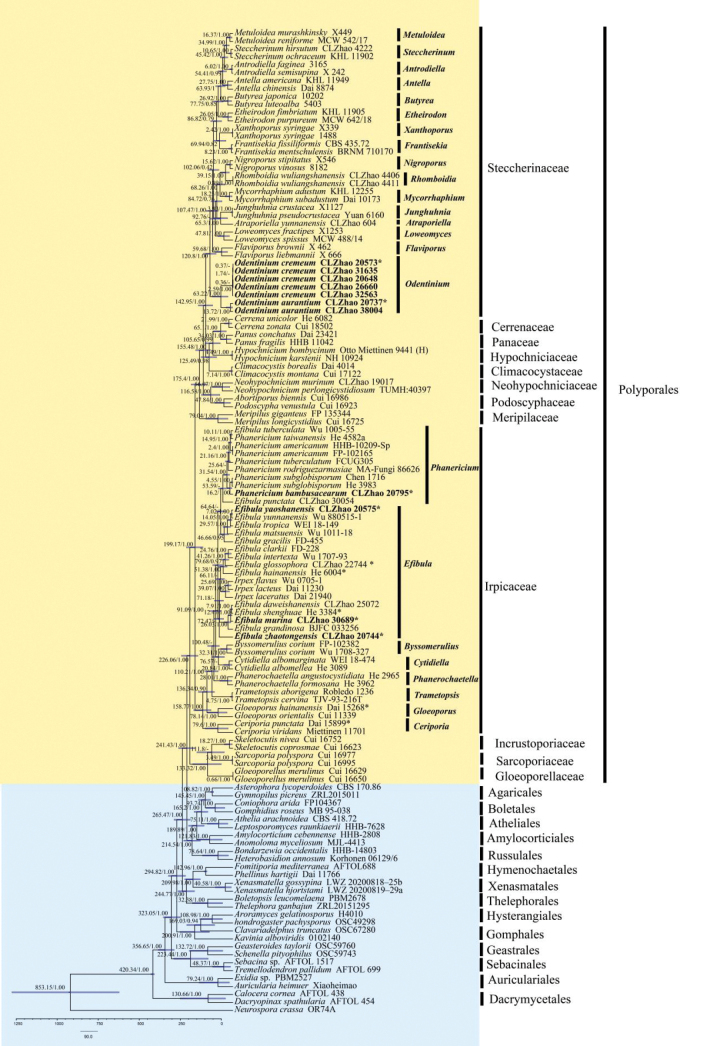
Divergence time estimation of families within *Polyporales* based on Bayesian evolutionary analysis sampling trees using the combined ITS, nLSU, mtSSU, *rpb*2, and *tef*1-α sequence dataset. Posterior probabilities are not less than 0.80, and the mean ages of each node are annotated. The 90% highest posterior density intervals of divergence time estimates are indicated by horizontal bars.

The combined ITS, nLSU, mtSSU, *rpb*2, and *tef*1-α dataset included 124 collections, of which 95 specimens belonged to *Polyporales*. This dataset resulted in a concatenated alignment of 6655 characters, with GTR+I+G identified as the best-fit evolutionary model. Chain convergence was indicated by an effective sample size (ESS) of 493. The results (Fig. 4) showed that the main clade of *Irpicaceae* emerged with a mean stem age of 199.17 Mya (95% HPD: 157.03–244.6 Mya). The genus *Efibula* formed distinct clades and was closely related to *Irpex* and *Phanericium*. Furthermore, the main clade of *Steccherinaceae* emerged with a mean crown age of 142.95 Mya (95% HPD: 106.63–182.43 Mya), followed by the genus *Odentinium*, which was estimated to have emerged with a mean crown age of 120.8 Mya (95% HPD: 88.28–156.14 Mya).

### ﻿Taxonomy

#### ﻿ Steccherinaceae

Taxon classificationAnimaliaPolyporalesSteccherinaceae

Parmasto, Consp. System. Corticiac. (Tartu): 169 (1968)

D3E82B0D-665E-57A8-A09B-409CFF6FB247

81420

##### Type genus.

*Steccherinum* Gray

##### Description.

***Basidiomata*** effused, effused-reflexed, or pileate, white or yellow to brown in various hues. ***Hymenial surface*** smooth, granular, aculeate, or poroid. context leathery to corky, whitish to pallid, more or less distinctly dimitic with skeletals. ***Hyphal system*** monomitic; generative hyphae branched, septate, or with clamp connections. ***Cystidia*** lacking or present, thin- to thick-walled. ***Basidia*** clavate or slightly multiform, 2–4-spored. ***Basidiospores*** cylindrical, ellipsoid, or subglobose, smooth, colorless, IKI– (Maas Geesteranus 1971; Liu and Dai 2021; Du et al. 2022).

##### Notes.

The family *Steccherinaceae* was typified by the genus *Steccherinum* (1968). It belongs to the residual polyporoid clade of *Polyporales* and represents a distinct and well-defined group based on phylogenetic evidence (Binder et al. 2013; Miettinen and Ryvarden 2016). *Steccherinaceae* includes around 24 genera according to previous studies and shows highly variable morphological and anatomical features (Zmitrovich 2018; Cao et al. 2021; He et al. 2024). In the present study, one new genus, *Odentinium*, is proposed in *Steccherinaceae* based on morphological and molecular evidence.

#### ﻿ Odentinium

Taxon classificationAnimaliaPolyporalesIrpicaceae

Y.L. Deng & C.L. Zhao gen. nov.

54E650FA-F6B4-581D-9CB4-D4C06CBA3681

856157

##### Etymology.

*Odentinium* (Lat.): referring to the odontioid hymenial surface.

##### Type species.

*Odentinium cremeum* Y.L. Deng & C.L. Zhao, sp. nov.

##### Description.

***Basidiomata*** annual, resupinate, leathery. ***Hymenial surface*** odontioid, aculei 3–6 per mm, the length of aculei up to 0.1 mm, cream to pale yellow. ***Hyphal system*** monomitic; generative hyphae with clamp connections, colorless, thin-walled, smooth, moderately branched. ***Cystidia*** numerous, thick-walled, cylindrical, strongly encrusted. ***Cystidioles*** absent. ***Basidia*** clavate, with four sterigmata and a basal clamp connection. ***Basidiospores*** ellipsoid, colorless, thin-walled, smooth.

##### Notes.

Westphalen et al. (2021) provided comprehensive morphological and phylogenetic analyses of hydnoid species in the family *Steccherinaceae*, identifying four genera, *Cabalodontia* Piatek, *Etheirodon*, *Metuloidea*, and *Steccherinum*. In the present study, the novel genus *Odentinium* is proposed based on morphological characteristics and phylogenetic analyses inferred from the combined ITS+nLSU+mtSSU+*rpb*2+*tef*1-α sequence dataset (Fig. 2). Six wood-inhabiting fungal specimens from Southwest China formed a distinct clade with strong support within *Steccherinaceae* in the phylogenetic analyses. Morphologically, these six specimens have soft corky to leathery basidiomata with odontioid hymenial surfaces that are cream to pale yellow and cover the aculei, a monomitic hyphal system, thin-walled generative hyphae with clamp connections, numerous thick-walled cylindrical cystidia, thin-walled clavate basidia with four sterigmata and a basal clamp connection, and thin-walled ellipsoid to globose basidiospores. These characteristics distinguish *Odentinium* from known genera in *Steccherinaceae*. Therefore, *Odentinium* is proposed as a new genus based on morphological characteristics and phylogenetic analyses.

#### ﻿ Odentinium aurantium

Taxon classificationAnimaliaPolyporalesIrpicaceae

Y.L. Deng & C.L. Zhao sp. nov.

264A7547-21DB-5DCB-8D22-93192E33C6CA

856159

Figs 5, 6

##### Diagnosis.

*Odentinium aurantium* is characterized by soft corky basidiomata, cream to pale yellow hymenial surface with odontioid, clavate basidia clavate and globose basidiospores (3–4 × 3–3.5 µm).

##### Etymology.

*Aurantium* (Lat.): referring to the pale yellow hymenial surface.

##### Type.

CHINA • Yunnan Province, Zhaotong, Qiaojia County, Yaoshan Town, Yaoshan National Nature Reserve, GPS coordinates: 27°08'N, 103°09'E, altitude 2220 m asl., on the fallen angiosperm branch, leg. C.L. Zhao, 23 August 2020, CLZhao 20737 (SWFC).

##### Description.

***Basidiomata*** annual, resupinate, soft corky when fresh, hard corky when dry, up to 5.5 cm long, 1.5 cm wide, and 0.2 mm thick. ***Hymenial surface*** odontioid, aculei 4–6 per mm, the length of aculei up to 0.1 mm, cream when fresh, cream to pale yellow upon drying. ***Sterile margin*** cream, thin, up to 1 mm. ***Hyphal system*** monomitic; generative hyphae with clamp connections, colorless, thin-walled, smooth, moderately branched, loosely interwoven, 2–3.7 µm in diameter, IKI–, CB–; tissues unchanged in KOH. ***Cystidia*** numerous, thick-walled, cylindrical, strongly encrusted, 65–182 × 10–12.5 µm. ***Cystidioles*** absent. ***Basidia*** clavate, with 4 sterigmata and a basal clamp connection, 11–23 × 4–4.5 µm, smooth, thin-walled, basidioles dominant, in shape similar to basidia, but slightly smaller. ***Basidiospores*** globose, colorless, thin-walled, smooth, with one oil drop, IKI–, CB–, 3–4 × 3–3.5 µm, L = 3.43 µm, W = 3.26 µm, Q = 1.12 (n = 60/2).

**Figure 5.**
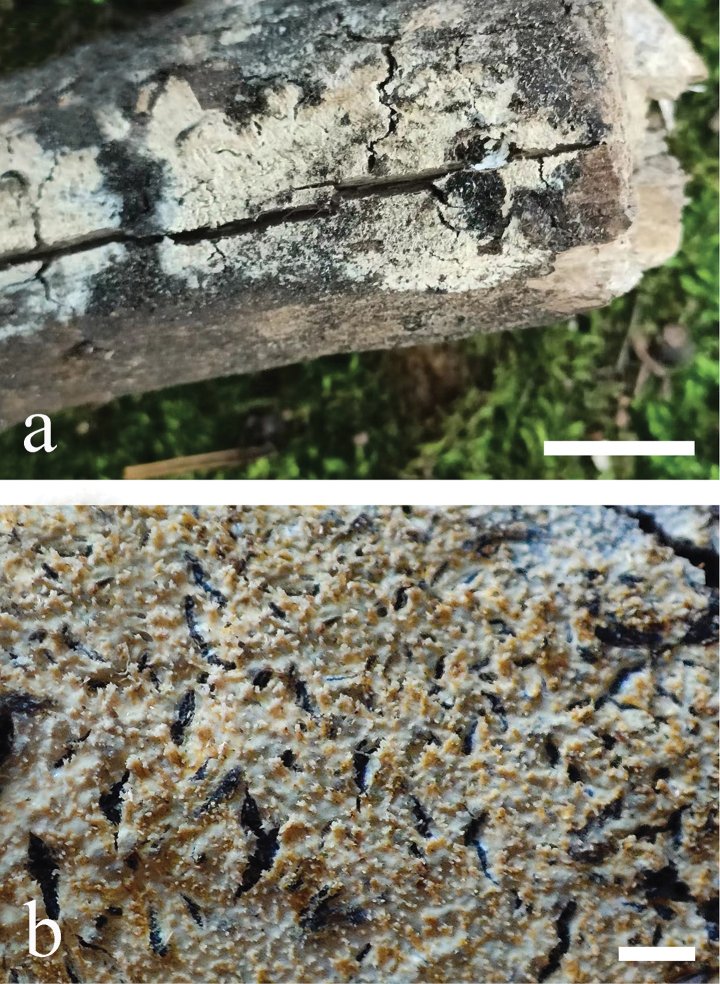
*Odentinium aurantium* (holotype, CLZhao 20737). **a** basidiomata. **b** macroscopic characteristics of hymenophore. Scale bars: 1 cm (**a**); 1 mm (**b**).

##### Additional specimen examined (Paratype).

CHINA • Yunnan Province, Zhaotong, Qiaojia County, Yaoshan Town, Yaoshan National Nature Reserve, GPS coordinates: 27°08'N, 103°09'E, altitude 2220 m asl., on the fallen angiosperm branch, leg. C.L. Zhao, 23 August 2020, CLZhao 38004 (SWFC).

**Figure 6.**
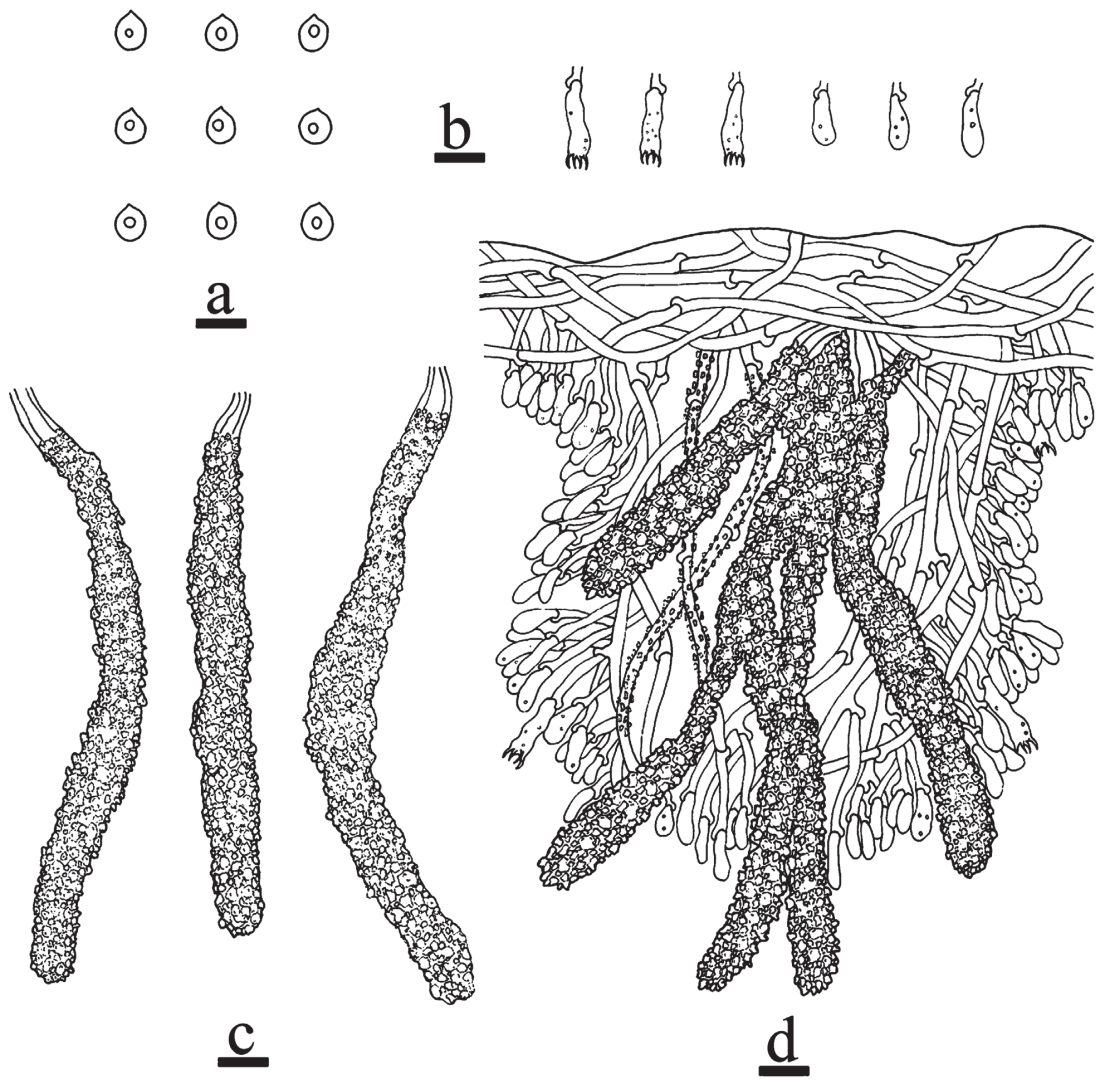
Microscopic structures of *Odentinium aurantium* (holotype, CLZhao 20737). **a** basidiospores. **b** basidia and basidioles. **c** cystidia. **d** a section of the hymenium. Scale bars: 5 µm (**a**); 10 µm (**b–d**).

##### Notes.

The topology of the tree (Fig. 2) inferred from ITS, nLSU, mtSSU, *rpb*2, and *tef*1-α sequence data showed that *Odentinium aurantium* was sister to *O. cremeum*; however, morphologically, *O. cremeum* can be distinguished by having larger aculei (3–4 per mm), a cream to slightly buff hymenial surface, and wider basidia (12–21 × 5–6 µm vs. 11–23 × 4–4.5 µm).

#### ﻿ Odentinium cremeum

Taxon classificationAnimaliaPolyporalesIrpicaceae

Y.L. Deng & C.L. Zhao sp. nov.

B7FC5CB5-9D89-5886-AD45-4290D326AAC8

856158

Figs 7, 8

##### Diagnosis.

*Odentinium cremeum* differs from other species by its cream to slightly buff hymenophore, clamed generative hyphae, and ellipsoid basidiospores (3.5–4.5 × 3–3.5 µm).

##### Etymology.

*Cremeum* (Lat.): referring to the species having cream-colored hymenial surface.

##### Type.

CHINA • Yunnan Province, Zhaotong, Qiaojia County, Yaoshan Town, Yaoshan National Nature Reserve, GPS coordinates: 27°08'N, 103°09'E, altitude 2220 m asl., on the fallen angiosperm branch, leg. C.L. Zhao, 22 August 2020, CLZhao 20573 (SWFC).

##### Description.

***Basidiomata*** annual, resupinate, leathery, up to 6.5 cm long, 3 cm wide, and 200 µm thick. ***Hymenial surface*** odontioid, aculei 3–4 per mm, the length of aculei up to 0.1 mm, slightly cream when fresh, turning to cream upon drying. ***Sterile margin*** white to slightly cream, thin, up to 1 mm. ***Hyphal system*** monomitic; generative hyphae with clamp connections, colorless, thin-walled, and strongly encrusted with crystals, moderately branched, interwoven, 1.5–4 µm in diameter, IKI–, CB–; tissues unchanged in KOH. ***Cystidia*** numerous, thick-walled, cylindrical, strongly encrusted entirely, 84–188 × 7–13 µm, cystidioles absent. ***Basidia*** clavate, with 4 sterigmata and a basal clamp connection, 12–21 × 5–6 µm, smooth, thin-walled, basidioles dominant, in shape similar to basidia, but slightly smaller. ***Basidiospores*** ellipsoid, colorless, thin-walled, smooth, with one oil drop, IKI–, CB–, 3.5–4.5 × 3–3.5 µm, L = 4.1 µm, W = 3.16 µm, Q = 1.29–1.33 (n = 150/5).

**Figure 7.**
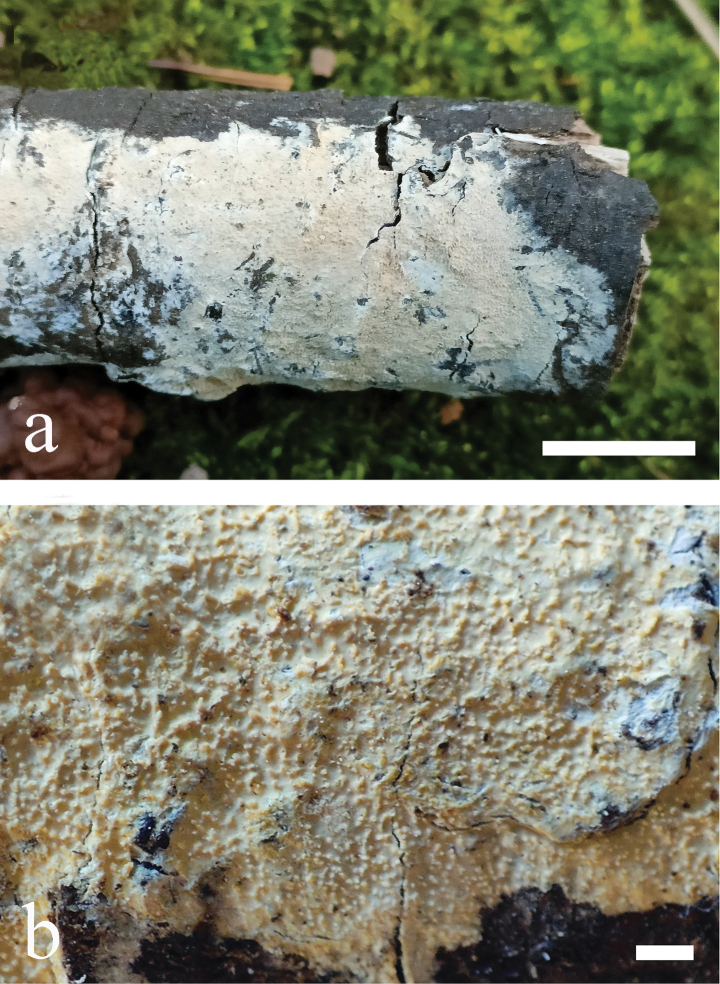
*Odentinium cremeum* (holotype, CLZhao 20573). **a** basidiomata. **b** macroscopic characteristics of hymenophore. Scale bars: 1 cm (**a**); 1 mm (**b**).

##### Additional specimens examined (Paratypes).

CHINA • Yunnan Province, Qujing, Qilin District, Cuishan Forest Park, 25°32'38.645"N, 103°41'28.860"E, , altitude 2245 m asl., on the fallen angiosperm branch, leg. C.L. Zhao, 5 November 2022, CLZhao 26660 (SWFC), Zhaotong, Wumeng Mountain National Nature Reserve, 27°19'24.215"N, 103°43'1.178"E, altitude 2200 m asl., on the fallen angiosperm branch, leg. C.L. Zhao, 26 August 2023, CLZhao 31635 (SWFC), 29 August 2023, CLZhao 32563 (SWFC), Zhaotong, Qiaojia County, Yaoshan Town, Yaoshan National Nature Reserve, GPS coordinates: 27°08'N, 103°09'E, altitude 2220 m asl., on the fallen angiosperm branch, leg. C.L. Zhao, 22 August 2020, CLZhao 20648 (SWFC).

**Figure 8.**
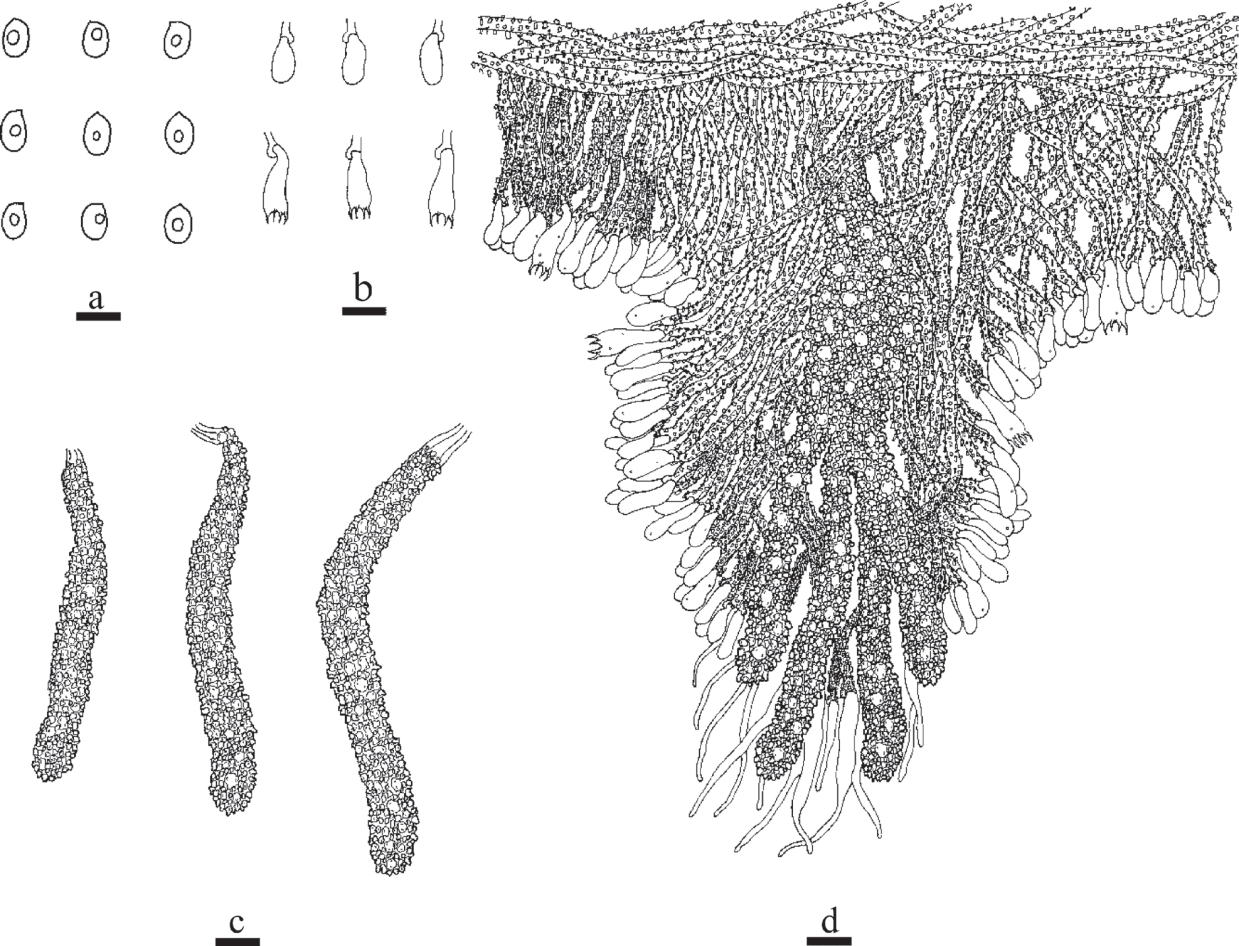
Microscopic structures of *Odentinium cremeum* (holotype, CLZhao 20573). **a** basidiospores. **b** basidia and basidioles. **c** cystidia. **d** a section of the hymenium. Scale bars: 5 µm (**a**); 10 µm (**b–d**).

##### Notes.

The phylogenetic analysis (Fig. 3) showed that *Odentinium cremeum* was sister to *O. aurantium*. However, morphologically, *O. aurantium* can be distinguished by a cream to pale yellow odontioid hymenial surface, aculei 4–6 per mm, narrower clavate basidia (11–23 × 4–4.5 µm vs. 12–21 × 5–6 µm), and globose basidiospores.

#### ﻿ Irpicaceae

Taxon classificationAnimaliaPolyporalesIrpicaceae

Spirin & Zmitr.

05E1822A-709F-57A8-94F5-A4C67D55D2A5

##### Type genus.

*Irpex* Fr. 1825.

#### ﻿ Efibula

Taxon classificationAnimaliaPolyporalesIrpicaceae

Sheng H. Wu

F3E78D6F-7B25-50C4-97F7-C4750E3C5AF9

25473

##### Type species.

*Efibula tropica* Sheng H. Wu

##### Description.

***Basidiomata*** annual, resupinate, adnate, at first orbicular and then becoming confluent. ***Hymenial surface*** white, whitish buff to “Amber,” thinning out indeterminately, sometimes pruinose under the lens. ***Hyphal system*** monomitic; generative hyphae simple-septate, smooth, thin- to slightly thick-walled. ***Cystidia*** absent or present, rarely fusiform cystidia present in the hymenium. ***Basidia*** cylindrical to clavate with a stalk-like base, sometimes slightly constricted without a basal clamp, producing four sterigmata. ***Basidiospores*** broadly ellipsoid, smooth, thin-walled (Chen et al. 2021, 2022; Li et al. 2022).

##### Notes.

The genus *Efibula* was circumscribed by Wu (1990), with *Efibula tropica* Sheng H. Wu designated as the type species (Wu 1990). *Efibula* was traditionally classified in the family *Phanerochaetaceae* (Kirk et al. 2008). With revisions to the family-level classification of the order *Polyporales*, phylogenetic analyses support the placement of *Efibula* in the family *Irpicaceae* (Justo et al. 2017; Chen et al. 2021; Osman and El-Nuby 2023; Wang et al. 2023). Although *Efibula* and *Phanericium* are not easy to separate morphologically, they are genetically distinct. In general, *Efibula* species have a more compact texture with a dense subiculum that is not always clearly distinguished from the subhymenium (Larsson et al. 2025). In this study, three new species (*E. murina*, *E. yaoshanensis*, and *E. zhaotongensis*) are introduced based on morphological characters and multigene phylogenetic evidence. Comparative characteristics of *Efibula* species are presented in Table 2 to establish taxonomic differentiation for the newly proposed species.

**Table 2.** A morphological comparison species of *Efibula* and *Phanericium*.

Species	Type locality	Basidiomata	Generative hyphae	Cystidia and cystidoles	Basidia (µm)	Basdiospores (µm)	Host trees	References
*Efibula aurata*	Union of South Africa	Broadly effuse	Simple septate, thin-walled	Absent	Clavate to broadly clavate, 25–40 × 6–8.5 µm	Ellipsoid, 7.5–9 × 4.5–5.5 µm	On unidentified wood	Zmitrovich et al. (2006)
*E. avellanea*	France	Resupinate, smooth	Simple septate, thin- to slightly thick-walled	Absent	Narrowly clavate, 25–30 × 4–5 µm	Ellipsoid, 5–7 × 2.5–3.5 µm	On *Quercus*	Wu (1990)
*E. bubalina*	Canary Islands	Broadly effuse	Simple septate, slightly thick-walled	Absent	Clavate, 30–42 × 7–9 µm	Ellipsoid to broadly ellipsoid, 8.5–9.5 × 5.5–6.5 µm	On deciduous wood	Zmitrovich et al. (2006)
*E. candidissima*	China	Resupinate, smooth	Simple septate, thin-walled	Absent	Clavate, 20–25 × 4–6 μm	ellipsoid to oblong ellipsoid, 4.8–5.8 × 3.3–4 μm	On rotten angiosperm wood	Zhang et al. (2025)
*E. clarkii*	Unites States of America	Resupinate, slightly tuberculate	Simple septate, thin-walled	Absent	Clavate, 25–39 × 5–7.5 μm	Oblong to ellipsoid, 6.0–7.0 × 3.0–3.5	On fallen *Quercus* sp. branch	Floudas and Hibbett (2015)
*E. cordylines*	New Zealand	Effused, smooth	Simple septate, thin- to thick-walled	Absent	Clavate, 25–35(–45) × 5–6 µm	Broadly ellipsoid to ellipsoid, 6–7.5 × 3.5–4.5	On hardwood branches	Zmitrovich et al. (2006)
*E. corymbata*	New Zealand	Resupinate, smooth	Simple septate, thin-walled	Absent	Clavate, 35–40 × 6.5–8.5 µm	Narrowly ellipsoid, 8–9.5 × 3.5–4.5 µm	On hardwood branches	Zmitrovich et al. (2006)
*E. cremea*	China	white to cream, grandinioid	Simple septate, thin- to thick-walled	Absent	Clavate, 17.5–25 × 5–7.5 µm	Ellipsoid, 4.7–5.7 × 3.3–4	On fallen angiosperm branch	Li et al. (2025a)
*E. daweishanensis*	China	Resupinate, grandinoid	Simple septate, thin-walled	Absent	Clavate, 14–19 × 5–6.5 µm	Elliposoid, 6–7.5 × 3.5–4.8	On fallen angiosperm branch	Dong et al. (2024)
*E. deflectens*	Finland	Resupinate, effused, smooth, or warted with small papillae	Simple septate, thin-walled	Absent	Clavate, 25–35 × 3–5 µm	Ellipsoid, 4–5 × 2.5–3 µm	on *Picea abies* and manufactured wood.	Wu (1990)
*E. glossophora*	China	Resupinate, smooth	Simple septate, thin- to slightly thick-walled	Absent	Long clavate, 21.5–26.7 × 5.7–7.4 µm	Ellipsoid, 3.8–6 × 2.6–3.7 µm	On fallen angiosperm branch	Gu et al. (2025)
*E. gracilis*	Unites States	Resupinate, smooth	Simple septate, thin-walled	Absent	Cylindrical to clavate, 17–30 × 5–6.5 µm	Ellipsoid to oblong, 5.5–7 × 3.3–4	On fallen branch	Floudas and Hibbett (2015)
*E. grandinosa*	China	Resupinate, grandinioid	Simple septate, thin- to slightly thick-walled		Clavate, 36–43 × 5–7 µm	Ellipsoid, 6–6.8 × 3.7–4 µm	On dead angiosperm branch	Li et al. (2022)
*E. hainanensis*	China	Resupinate, smooth	Simple septate, thin- to slightly thick-walled	Cystidia subfusiform to subcylindrical	Clavate, 15–26 × 4–6 µm	Ellipsoid to broadly ellipsoid, 4.2–5.5 × 2.8–3.2	On dead liana	Li et al. (2022)
*E. intertexta*	China	Effused, smooth	Simple septate, thin- to slightly thick-walled	Absent	Narrowly clavate, 30–35 × 4.5–5 μm	Cylindrical, 5.6–6.4 × 2.2–2.6 μm	On angiosperm branch	Chen et al. 2021
*E. matsuensis*	China	Effused, smooth	simple septate, thick walled	Absent	Clavate, 18–25 × 6.5–8 μm	Ellipsoid to cylindrical, 7.4–8.6 × 3.8–4.4 μm	On angiosperm branch,	Chen et al. (2021)
***E. murina***	**China**	**Resupinate, tuberculate**	**Simple septate, thin- to slightly thick-walled**	**Absent**	**Clavate, 15–24 × 4–6 µm**	**Ellipsoid, 4–5.5 × 2.5–3 µm**	**On fallen angiosperm branch**	**Present study**
*E. punctata*	China	Resupinate, smooth	Simple septate, thin-walled	Cystidoles subfusiform	Subcylindrical to subclavate, 11.8–19.4 × 3.7–5.9 µm	Ellipsoid, 4.3–6.2 × 2.2–3.3	On fallen angiosperm branch	Dong et al. (2024)
*E. shenghuae*	China	Smooth to grandinioid	Simple septate, slightly thick-walled	Absent	Clavate, 23–38 × 4.5–7 µm	Oblong ellipsoid, 6–6.5 × 3–3.5	On dead branch of *Quercus*	Li et al. (2022)
*E. tropica*	China	Effused, smooth	Simple septate, thin-walled	Cystidioles fusiform	Clavate to narrowly clavate, 27–40 × 5.5–8 μm	Broadly ellipsoid, 6.4–7.7 × 3.7–4.4 μm	On decaying angiosperm branch	Chen et al. (2021)
*E. turgida*	China	Resupinate, smooth	Simple septate, thin-walled	Absent	Clavate to subclavate, 26–30 × 6.5–7 μm	Cylindrical, 6.6–8.2 × 3.3–3.9 μm	On angiosperm branch	Chen et al. (2021)
*E. verruculosa*	Tanzania	Resupinate, grandinioid to odontoid	Simple septate, thin-walled	Absent	Clavate, 15–20 × 5 µm	Ellipsoid to subcylindrical, 4–4.5 × 2–2.5 µm	—	Kotiranta and Saarenoksa (1993)
***E. yaoshanensis***	**China**	**Resupinate, smooth**	**Simple septate, thin-walled**	**Absent**	**Long clavate, 20–27 × 4–6 µm**	**Ellipsoid, 5–6.5 × 3–4 µm**	**On fallen angiosperm branch**	**Present study**
*E. yunnanensis*	China	Resupinate, smooth	Simple septate, thin-walled	Absent	Clavate, 25–31 × 6–7.5 µm	Ellipsoid, 5.5–7.5 × 3.6–4.5	On fallen branch of angiosperm	Ma et al. (2020)
***E. zhaotongensis***	**China**	**Resupinate, grandinioid**	**Clamped, thin-walled**,	**Absent**	**Clavate, 17–25 × 4–5 µm**	**Cylindrical, 4–5.5 × 2–3.5 µm**	**On fallen angiosperm branch**	**Present study**
*P. americanum*	Unites States	Resupinate, smooth to reticulate	Clamped, thin-walled	Absent	Cylindrical to clavate, 20–32 × 5–8 µm	Ellipsoid to cylindrical, 5.3–6.5 × 3–3.8 µm	—	Floudas and Hibbett (2015)
***P. bambusacearum***	**China**	**Resupinate, smooth**	**Simple septate, thin-walled**	**Absent**	**Long clavate, 41–60 × 6–9 µm**	**Subglobose, 4.5–7 × 3.5–5 µm**	**On the dead bamboo**	**Present study**
*P. gemellum*	Norway	Effused, smooth	Simple septate, thin- to slightly thick-walled	Absent	Long clavate, 19–40 × 5.2–7.4 μm	Narrowly ellipsoid to ellipsoid, 5.2–7.8 × 3.1–4.7 μm	On angiosperm wood	Larsson et al. (2025)
*P. rodriguezarmasiae*	Spain	Resupinate, effuse, smooth to tuberculate	Clamped, thin-walled	Absent	Claviform, 35–48 × 6–8 µm	Ellipsoid, 6–7 × 4–5 µm	On *Euphorbia regis-jubae*	Boonmee et al. (2021)
*P. subglobisporum*	China	Effused, smooth	Simple septate, thin- to slightly thick-walled	Absent	Clavate, 30–40 × 6.5–8 μm	Broadly ellipsoid to subglobose, 6.4–8.1 × 4.5–5.8 μm	On angiosperm branch	Chen et al. (2021)
*P. taiwanense*	China	Resupinate, smooth	Simple septate, thin- to slightly thick-walled	Absent	Clavate to subcylindrical, 24–44 × 6–8 µm	Broadly ellipsoid to ovoid, 5.8–6.5 × 4–4.5 µm	On dead angiosperm branch	Li et al. (2022)
*P. tuberculatum*	Finland	Effused, smooth	Simple septate, thin- to slightly thick-walled	Absent	Narrowly clavate, 25–35 × 4–5 µm	Ellipsoid, 5–7 × 3–4 µm	On angiosperm wood and gymnosperm	Chen et al. (2021)

#### ﻿ Efibula murina

Taxon classificationAnimaliaPolyporalesIrpicaceae

Y.L. Deng & C.L. Zhao sp. nov.

66EB241B-6559-5628-B2FE-CBB90D960817

854028

Figs 9, 10

##### Diagnosis.

The new species is distinguished from all other *Efibula* species by its cracked, slightly gray to gray hymenophore with tuberculate, generative hyphae bearing simple septa, clavate basidia, and ellipsoid basidiospores (4–5.5 × 2.5–3 µm).

##### Etymology.

*Murina* (Lat.): referring to the species having furry mouse-like hymenial surface.

##### Type.

CHINA • Yunnan Province, Dehong, Yingjiang County, Tongbiguan Provincial Reserve, GPS coordinates: 24°69'N, 97°94'E, altitude 2500 m asl., on fallen angiosperm branch, leg. C.L. Zhao, 20 July 2023, CLZhao 30689 (SWFC).

##### Description.

***Basidiomata*** annual, resupinate, closely adnate, membranous, up to 8 cm long, 1 cm wide, and 200 µm thick. ***Hymenial surface*** tuberculate, slightly gray when fresh, gray upon drying, cracked; margin slightly gray, up to 1.5 mm. ***Hyphal system*** monomitic, generative hyphae bearing simple septa, IKI–, CB–; tissues unchanged in KOH. ***Subiculum*** generative hyphae colorless, thin- to slightly thick-walled, smooth, moderately branched, loosely interwoven, 1.5–4 µm in diameter; subhymenium thin, generative hyphae colorless, thin- to slightly thick-walled, smooth, moderately branched, interwoven, 1.5–3.5 µm in diameter. ***Cystidia*** and cystidioles absent. ***Basidia*** clavate, with four sterigmate, with a simple septum at the base, 15–24 × 4–6 µm, usually with some small oily drops, smooth, thin-walled, basidioles dominant, in shape similar to basidia, but slightly smaller. ***Basidiospores*** ellipsoid, colorless, thin-walled, smooth, usually with some small oily drops, IKI–, CB–, 4–5.5(–6) × 2.5–3 µm, L = 4.76 µm, W = 2.9 µm, Q = 1.63–1.64 (n = 60/2).

**Figure 9.**
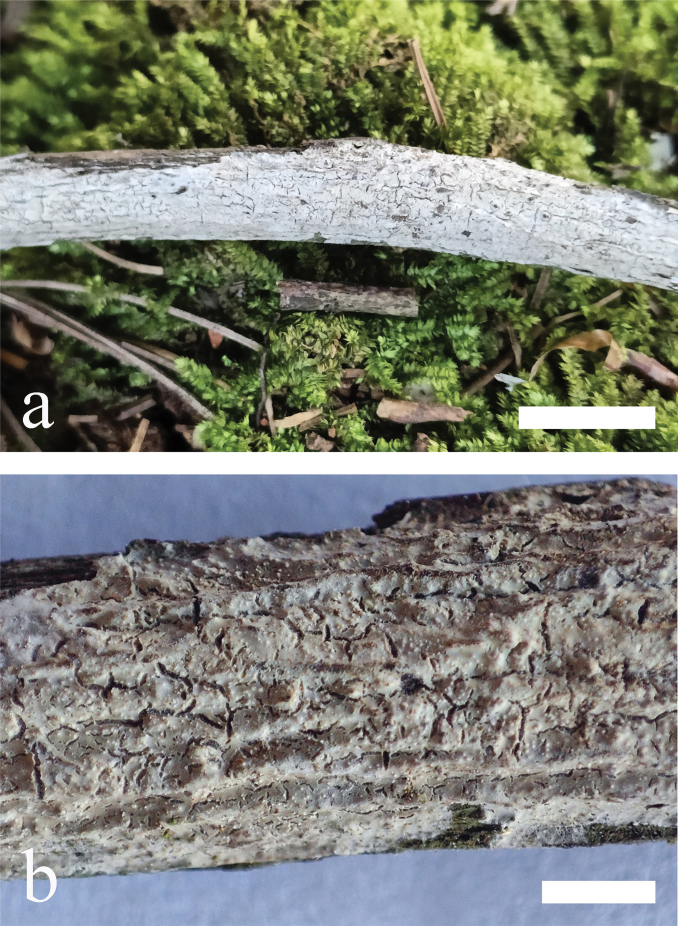
*Efibula murina* (holotype, CLZhao 30689). **a** basidiomata. **b** macroscopic characteristics of hymenophore. Scale bars: 1 cm (**a**); 1 mm (**b**).

##### Additional specimens examined (Paratypes).

CHINA • Yunnan Province, Xishuangbanna, Jinghong City, Rubber Plantation, GPS coordinates: 21°90'N, 100°76'E, altitude 552.7 m asl., on the fallen angiosperm branch, leg. C.L. Zhao, 25 January 2024, CLZhao 35686, CLZhao 35695, CLZhao 35707 (SWFC).

**Figure 10.**
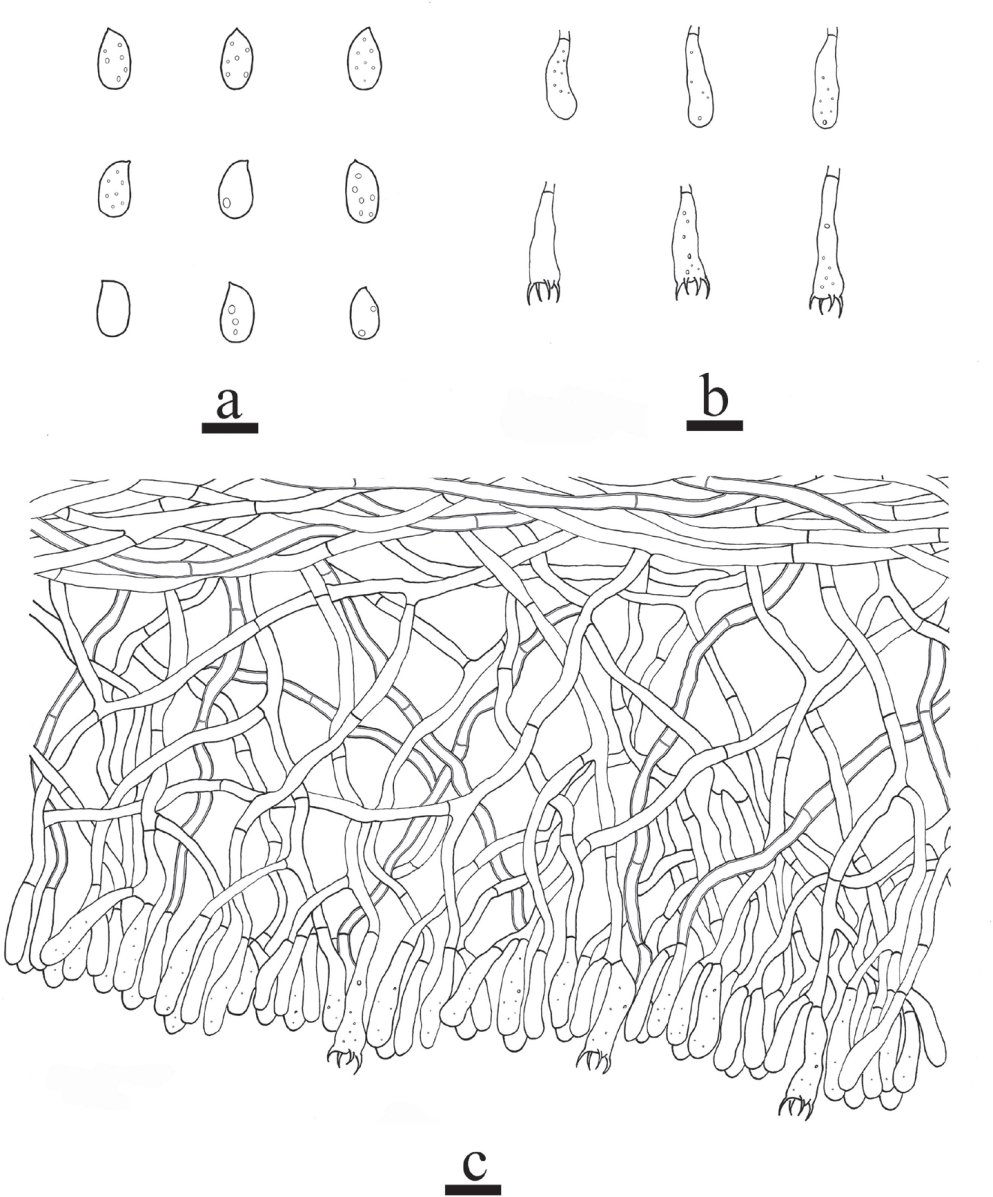
Microscopic structures of *Efibula murina* (holotype, CLZhao 30689). **a** basidiospores. **b** basidia and basidioles. **c** a section of the hymenium. Scale bars: 5 µm (**a**); 10 µm (**b, c**).

##### Notes.

The phylogenetic analyses (Figs 4, 5) showed that the new species *Efibula murina* is grouped within the genus *Efibula* and is closely related to *Efibula grandinosa* Yue Li & S.H. He and *E. shenghuae* Yue Li & S.H. He. However, morphologically, *E. grandinosa* differs from *E. murina* by having a pale orange to grayish orange, grandinioid hymenial surface with projecting hyphal pegs, slightly darkening in KOH, larger basidia (36–43 × 5–7 µm vs. 15–24 × 4–6 µm), and larger basidiospores with an apiculus (6–6.8 × 3.7–4 µm vs. 4–5.5 × 2.5–3 µm; Li et al. 2022). The species *E. shenghuae* can be easily distinguished from *E. murina* by an uncracked smooth to grandinioid orange-white to pale orange hymenophore with irregular and scattered granules, longer basidia (23–38 × 4.5–7 µm vs. 15–24 × 4–6 µm), and larger oblong ellipsoid basidiospores with an apiculus (6–6.5 × 3–3.5 µm vs. 4–5.5 × 2.5–3 µm; Li et al. 2022).

#### ﻿ Efibula yaoshanensis

Taxon classificationAnimaliaPolyporalesIrpicaceae

Y.L. Deng & C.L. Zhao sp. nov.

B6E50500-CD9E-5FF5-999E-D5BA210487C3

854029

Figs 11, 12

##### Diagnosis.

*Efibula yaoshanensis* differs from other species in the genus by cream, brownish-orange to cinnamon-buff hymenial surface, thin-walled generative hyphae bearing simple septa, and ellipsoid basidiospores (5–6.5 × 3–4 µm).

##### Etymology.

*Yaoshanensis* (Lat.): referring to the locality (Yaoshan) of the type specimen.

##### Type.

CHINA • Yunnan Province, Zhaotong, Qiaojia County, Yaoshan Town, Yaoshan National Nature Reserve, GPS coordinates: 27°08'N, 103°09'E, altitude 2220 m asl., on fallen angiosperm branch, leg. C.L. Zhao, 22 August 2020, CLZhao 20575 (SWFC).

**Figure 11.**
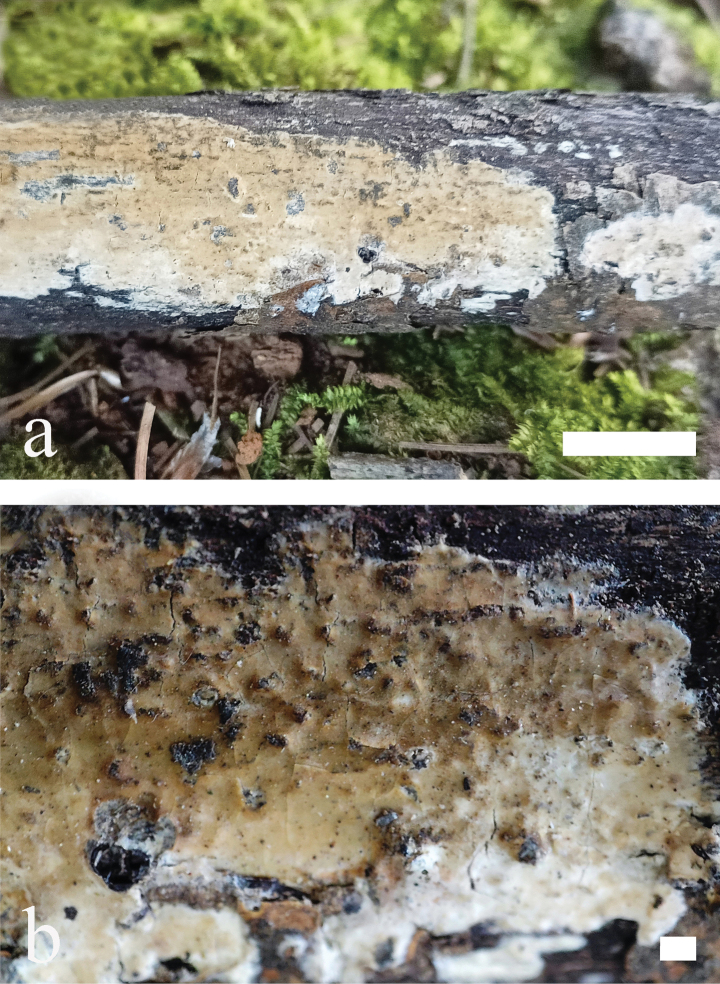
*Efibula yaoshanensis* (holotype, CLZhao 20575). **a** basidiomata. **b** macroscopic characteristics of hymenophore. Scale bars: 1 cm (**a**); 1 mm (**b**).

##### Description.

***Basidiomata*** annual, resupinate, closely adnate, membranous to subceraceous, up to 7 cm long, 1.8 cm wide, and 200 µm thick. ***Hymenial surface*** smooth, cream when fresh, brownish-orange to cinnamon-buff upon drying; margin thinning out, white to cream, up to 1 mm. ***Hyphal system*** monomitic; generative hyphae bearing simple septa, colorless, thin-walled, slightly encrusted with crystals on some hyphae, 1.8–3.2 µm in diameter; IKI–, CB–; tissues unchanged in KOH. ***Cystidia*** and cystidioles absent. ***Basidia*** long clavate, four sterigmate, with a simple septum at the base, 20–27 × 4–6 µm, smooth, thin-walled, basidioles dominant, in shape similar to basidia, but slightly smaller. ***Basidiospores*** ellipsoid, colorless, thin-walled, smooth, IKI–, CB–, 5–6.5 × 3–4 µm, L = 5.56 µm, W = 3.21 µm, Q = 1.74 (n = 30/1).

**Figure 12.**
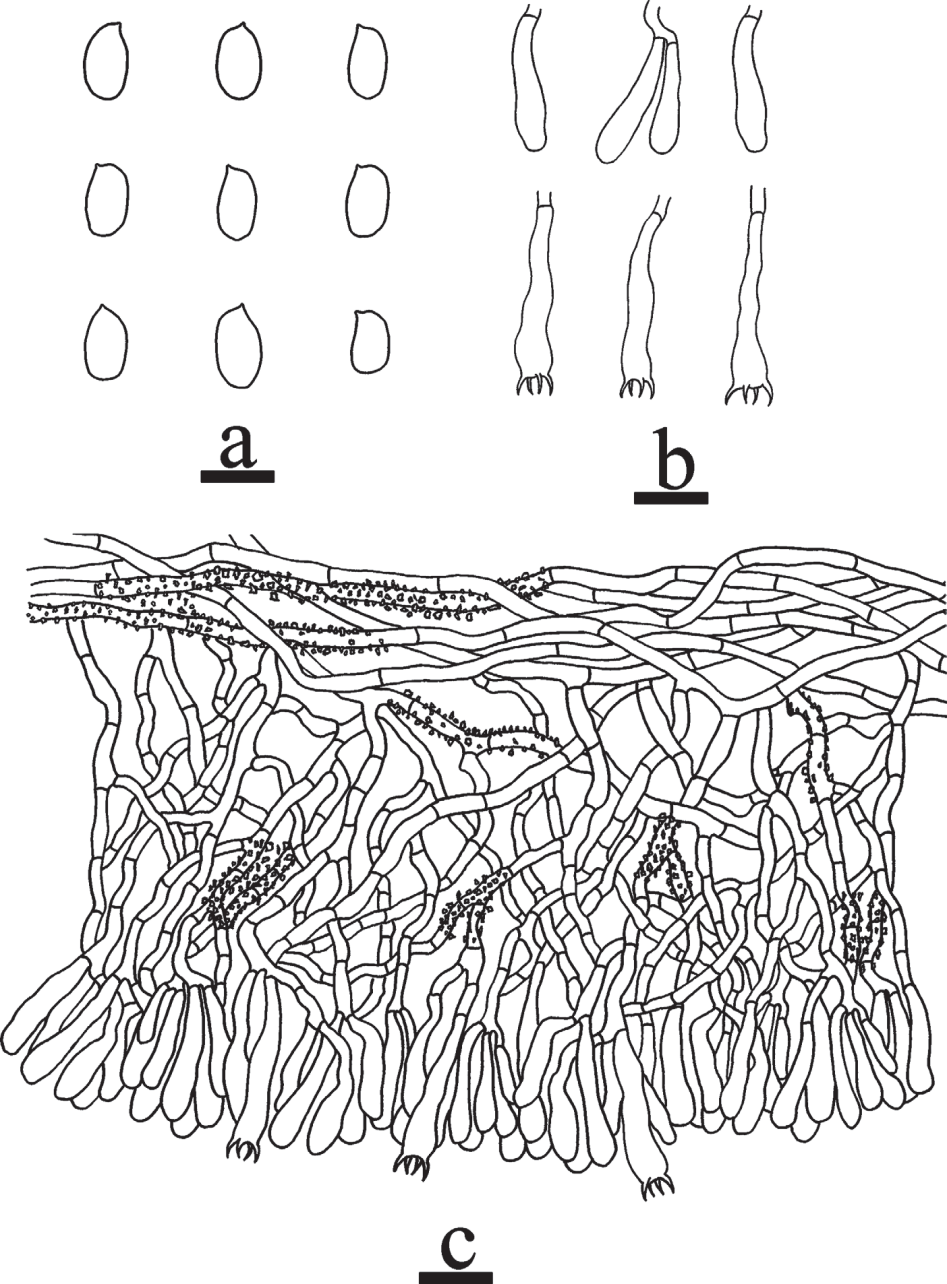
Microscopic structures of *Efibula yaoshanensis* (holotype, CLZhao 20575). **a** basidiospores. **b** basidia and basidioles. **c** a section of the hymenium. Scale bars: 5 µm (**a**); 10 µm (**b, c**).

##### Notes.

The phylogenetic analyses (Figs 4, 5) showed that the new species *Efibula yaoshanensis* was sister to *E. yunnanensis* C.L. Zhao. However, morphologically, *E. yunnanensis* differs from *E. yaoshanensis* by having a smooth, cream to pale brown hymenial surface (Ma et al. 2020).

#### ﻿ Efibula zhaotongensis

Taxon classificationAnimaliaPolyporalesIrpicaceae

Y.L. Deng & C.L. Zhao sp. nov.

F616F95F-DB2E-5DE6-88C9-E226162FA15C

856161

Figs 13, 14

##### Diagnosis.

*Efibula zhaotongensis* can be distinguished from other species by its white to slightly cream hymenial surface grandinioid, generative hyphae with clamp connections, cylindrical basidiospores (4–5.5 × 2–3.5 µm).

##### Etymology.

*Cremea* (Lat.): referring to the species having cream hymenial surface.

##### Type.

CHINA • Yunnan Province, Zhaotong, Qiaojia County, Yaoshan Town, Yaoshan National Nature Reserve, GPS coordinates: 27°08'N, 103°09'E, altitude 2220 m asl., on fallen angiosperm branch, leg. C.L. Zhao, 23 August 2020, CLZhao 20744 (SWFC).

##### Description.

***Basidiomata*** annual, resupinate, coriaceous, up to 9 cm long, 2 cm wide, and 300 µm thick in section. ***Hymenial surface*** grandinioid, white to slightly cream when fresh, cream upon drying; margin white to slightly cream, up to 1 mm. ***Hyphal system*** monomitic; generative hyphae with clamp connections, colorless, thin-walled, moderately branched, encrusted with crystals, 1.8–4.1 µm in diameter, IKI–, CB–; tissues unchanged in KOH. ***Subiculum*** generative hyphae dense, subparallel arrangement; subhymenium composed of strongly agglutinated vertical hyphae. ***Cystidia*** and cystidioles absent. ***Basidia*** clavate, with 4 sterigmata and a basal clamp connection, 17–25 × 4–5 µm, smooth, thin-walled, basidioles dominant, in shape similar to basidia, but slightly smaller. ***Basidiospores*** cylindrical, colorless, thin-walled, smooth, IKI–, CB–, 4–5.5 × 2–3.5 µm, L = 4.76 µm, W = 2.78 µm, Q = 1.72 (n = 60/2).

**Figure 13.**
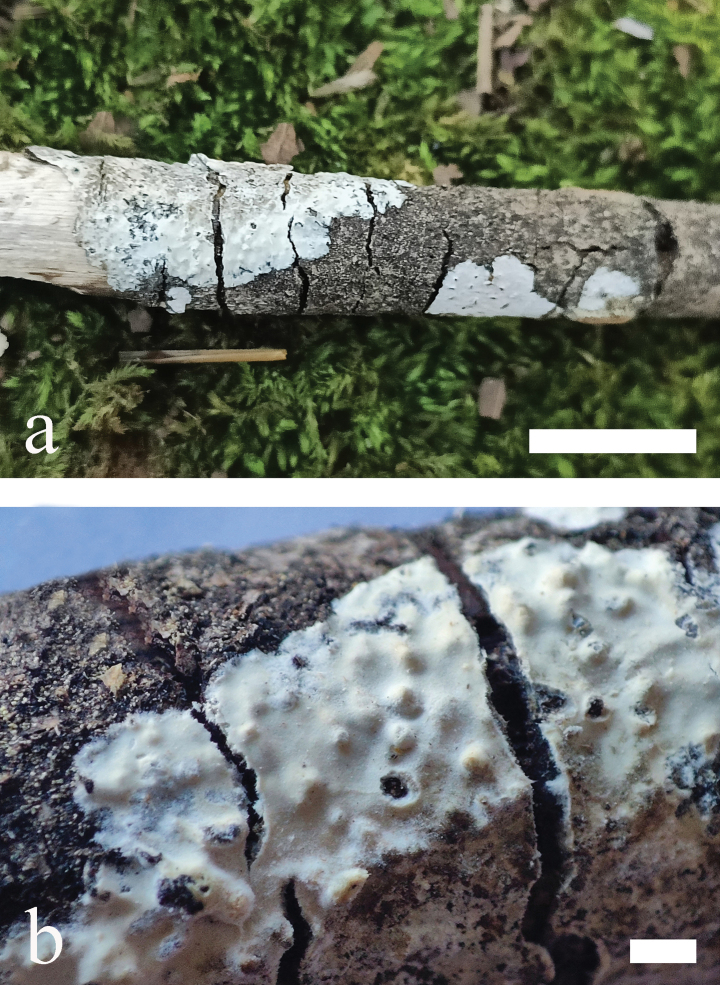
*Efibula zhaotongensis* (holotype, CLZhao 20744). **a** basidiomata. **b** macroscopic characteristics of hymenophore. Scale bars: 1 cm (**a**); 1 mm (**b**).

##### Additional specimen examined (Paratype).

CHINA • Yunnan Province, Zhaotong, Qiaojia County, Yaoshan Town, Yaoshan National Nature Reserve, GPS coordinates: 27°08'N, 103°09'E, altitude 2220 m asl., on fallen angiosperm branch, leg. C.L. Zhao, 23 August 2020, CLZhao 38003 (SWFC).

**Figure 14.**
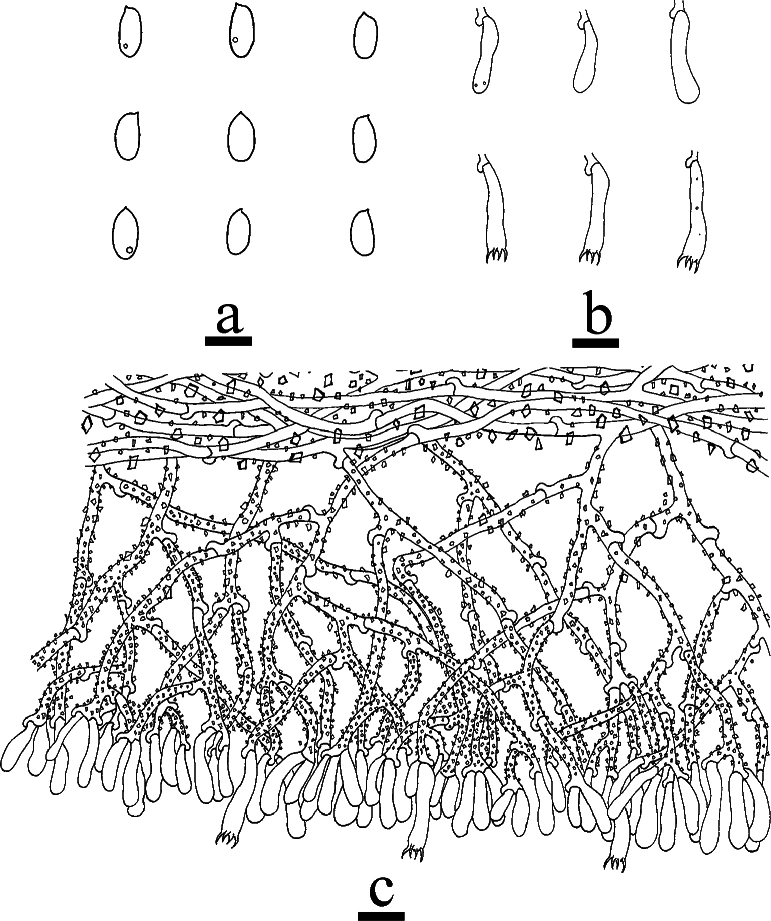
Microscopic structures of *Efibula zhaotongensis* (holotype, CLZhao 20744). **a** basidiospores. **b** basidia and basidioles. **c** a section of the hymenium. Scale bars: 5 µm (**a**); 10 µm (**b, c**).

##### Notes.

In this study, the phylogenetic analyses (Figs 3, 4) showed that the new species *Efibula zhaotongensis* is classified within the genus *Efibula* and is sister to *E. bubalina*. However, *E. bubalina* can be distinguished from *E. zhaotongensis* by having slightly thick-walled generative hyphae bearing simple septa, larger basidia (30–42 × 7–9 µm vs. 17–25 × 4–5 µm), and ellipsoid to broadly ellipsoid basidiospores (8.5–9.5 × 5.5–6.5 µm vs. 4–5.5 × 2–3.5 µm; Zmitrovich et al. 2006).

#### ﻿ Phanericium

Taxon classificationAnimaliaPolyporalesIrpicaceae

(Parmasto) K.H. Larss. & Spirin

31AE172B-4C70-592D-B321-87A124EB73E6

859316

##### Type species.

*Corticium tuberculatum* P. Karst.

##### Description.

***Basidiomata*** resupinate, effused, membranous, soft-ceraceous, whitish or with yellowish, orange, or pale brownish tints, hymenium smooth, margin thinning out, abrupt, arachnoid or fimbriate, context white. ***Hyphal system*** monomitic, septa without clamps or with occasional clamps on subicular hyphae, subicular hyphae thin- to slightly thick-walled, mostly parallel and extending horizontally over the substrate, sometimes strongly covered by grainy crystals, subhymenium thickening, rather dense, hyphae thin-walled, growing vertically. ***Cystidia*** absent. ***Basidia*** clavate to narrowly clavate, with four sterigmata. ***Basidiospores*** narrowly ellipsoid to ellipsoid to subglobose, smooth, thin-walled, without reaction to Melzer’s or Cotton Blue (Larsson et al. 2025).

##### Notes.

Recent phylogenetic analyses demonstrate that *Efibula* is non-monophyletic (Floudas and Hibbett 2015; Chen et al. 2021; Li et al. 2022). Recent studies confirm earlier observations and recover a strongly supported clade centered around *Corticium tuberculatum* (Larsson et al. 2025). Therefore, Larsson et al. (2025) introduced the new genus *Phanericium*, which is clearly separated from the core of *Efibula* around the type *E. tropica*. In this study, a new species, *Phanericium bambusacearum*, is proposed based on morphological characters and multigene phylogenetic evidence. Comparative characteristics of *Phanericium* species are presented in Table 2 to establish taxonomic differentiation for the newly proposed species.

#### ﻿ Phanericium bambusacearum

Taxon classificationAnimaliaPolyporalesIrpicaceae

Y.L. Deng & C.L. Zhao sp. nov.

A46625D2-849E-52BF-B491-8C0D2FD5F4DF

854027

Figs 15, 16

##### Diagnosis.

*Phanericium bambusacearum* differs from other species in the genus by cream, buff to pale-yellow, cracked hymenophore, generative hyphae bearing simple septa, long clavate basidia, and thin-walled, subglobose basidiospores (4.5–7 × 3.5–5 µm).

##### Etymology.

*Bambusacearum* (Lat.): refers to the species growing on bamboo.

##### Type.

CHINA • Yunnan Province, Zhaotong, Qiaojia County, Yaoshan Town, Yaoshan National Nature Reserve, GPS coordinates: 27°08'N, 103°09'E, altitude 2220 m asl., on the dead bamboo, leg. C.L. Zhao, 23 August 2020, CLZhao 20795 (SWFC).

##### Description.

***Basidiomata*** annual, resupinate, ceraceous, up to 7 cm long, 2.4 cm wide, and 250 µm thick. ***Hymenophore*** smooth, cream to buff when fresh, buff to pale-yellow upon drying, cracked; margin cream, up to 1 mm. ***Hyphal system*** monomitic; generative hyphae bearing simple septa, colorless, thin-walled, moderately branched, 1.8–3.2 µm in diameter, IKI–, CB–; tissues unchanged in KOH. ***Subiculum*** composed of a basal layer and a medullary layer, basal layer with dense texture encrusted with crystals, medullary layer with dense texture, hymenial layer slightly thickening, subhymenium with fairly dense texture. ***Cystidia*** and cystidioles absent. ***Basidia*** long clavate, smooth, thin-walled, with four sterigmata and a base simple septum, 41–60 × 6–9 µm, basidioles dominant, in shape similar to basidia, but slightly smaller. ***Basidiospores*** subglobose, colorless, thin-walled, smooth, IKI–, CB–, (4)4.5–7 × 3.5–5 µm, L = 6.03 µm, W = 4.47 µm, Q = 1.35 (n = 30/1).

**Figure 15.**
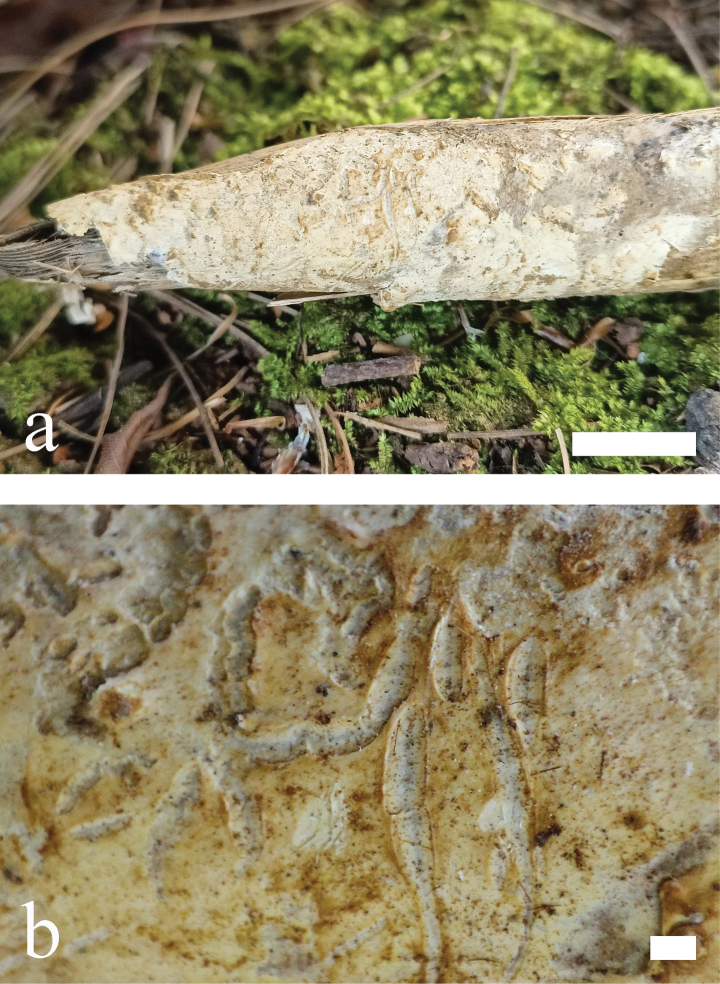
Basidiomata of *Phanericium bambusacearum* (holotype 20795). **a** basidiomata. **b** macroscopic characteristics of hymenophore. Scale bars: 1 cm (**a**); 1 mm (**b**).

**Figure 16.**
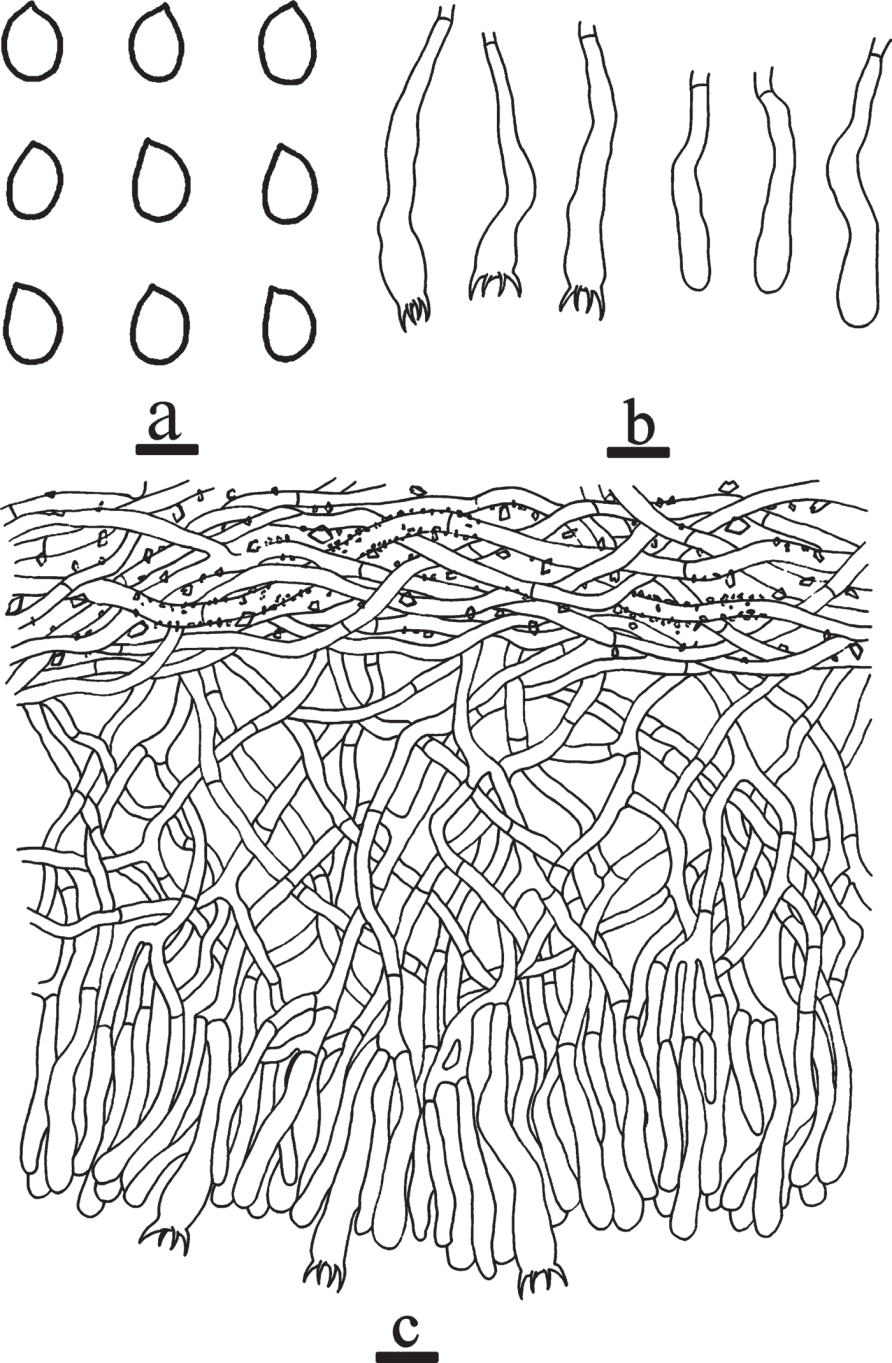
Microscopic structures of *Phanericium bambusacearum* (holotype 20795). **a** basidiospores. **b** basidia and basidioles. **c** a section of the hymenium. Scale bars: 5 µm (**a**); 10 µm (**b, c**).

##### Notes.

The phylogenetic analyses (Figs 3, 4) showed that the new species *Phanericium bambusacearum* is grouped within the genus *Phanericium* and is sister to *P. subglobisporum*. However, *Phanericium subglobisporum* can be delimited from *P. bambusacearum* by its uncracked hymenial surface, smaller clavate basidia (30–40 × 6.5–8 µm vs. 41–60 × 6–9 µm), and broadly ellipsoid to subglobose basidiospores (Chen et al. 2021).

## ﻿Discussion

With the rapid application of molecular technology, many new taxa of wood-inhabiting fungi have been continuously reported and recorded worldwide (Liu et al. 2022a, b; Wijayawardene et al. 2022; Jayawardena et al. 2023; Deng et al. 2024a, b; Dong et al. 2024; Yang et al. 2025). In addition, global studies have explored the diversity, ecology, and systematics of wood-inhabiting fungi, with variations in focus among the taxa studied (Wijayawardene et al. 2022; Jayawardena et al. 2023; Wijesinghe et al. 2025). The subtropical monsoon climate and high average annual precipitation favor the establishment of a diverse macrofungal community (Huang et al. 2023). Significant progress has been made in resolving phylogenetic relationships within wood-inhabiting fungi based on morphology, multilocus data, and genomic phylogenies (Cui et al. 2019; Chen et al. 2021; Liu et al. 2022a, b; 2023a, b, c; Hyde et al. 2024a, b; Yang et al. 2025).

*Polyporales* is a diverse group of *Agaricomycetes* that has received extensive attention and scientific study. In the dating analyses, divergence times of the studied taxa were estimated using crown ages, as proposed by Varga et al. (2019). Phylogenomic analyses by He et al. (2024) suggested that fungal orders diverged within a time range of 102–361 Mya. The divergence time of *Polyporales*, including *Irpicaceae* and *Steccherinaceae*, was estimated with a mean crown age of 241.43 Mya. Currently, large-scale phylogenetic reconstructions of *Polyporales* still suffer from missing data and limited taxon sampling, which influence inferences of backbone relationships. Therefore, further multi-marker sequencing of these taxa by other researchers is necessary.

*Irpicaceae* is one family within the phlebioid clade and includes species with poroid, irpicoid, or corticioid hymenial surfaces (Binder et al. 2013; Justo et al. 2017; Chen et al. 2020). Chen et al. (2021) identified two well-supported clades, *Ceriporia* and *Trametopsis*, for the first time. Most accepted genera are resolved as monophyletic groups, except *Ceriporia* and *Efibula* (Jia et al. 2014). The corticioid genus *Efibula* was segregated from *Phanerochaete* s.l. (Wu 1990) and was first incorporated into a concatenated 5.8S, nLSU, *rpb*1, and *rpb*2 dataset phylogeny, where it was recovered as monophyletic by Floudas and Hibbett (2015). Chen et al. (2021) and Luo et al. (2024), using combined ITS, 28S, *rpb*1, *rpb*2, and *tef*1-α datasets, showed that *Efibula* is closely related to *Phanerochaete* and *Irpex*. In the present study, based on ITS+nLSU phylogenetic analyses (Fig. 3), the classification of *Irpicaceae* showed that *Efibula* is closely related to *Irpex* and *Phanericium*. These genera cause white rot; however, morphological studies indicate that *Irpex* differs from *Efibula* by having resupinate or pileate basidiomata with smooth, poroid, or hydnoid hymenial surfaces (Chen et al. 2021). Morphologically, *Efibula* can be distinguished from *Phanericium* by its more compact texture with a dense subiculum that is not always clearly distinguished from the subhymenium (Larsson et al. 2025). Species of *Efibula* and *Phanericium* grow on different angiosperm and gymnosperm trees, causing white rot. The host trees of these fungi are summarized in Table 2. Most *Efibula* and *Phanericium* species grow on angiosperm wood, including the four new species reported in the present study. Moreover, the substrate of *Efibula aurata* (Bourdot & Galzin) Zmitr. & Spirin was recorded simply as wood, without specification of angiosperm or gymnosperm origin (Zmitrovich et al. 2006). *Efibula deflectens* (P. Karst.) Sheng H. Wu was collected on *Picea abies* and manufactured wood (Wu 1990).

The family *Steccherinaceae* was typified by the genus *Steccherinum* and belongs to the residual polyporoid clade of the order *Polyporales* (Basidiomycota). It represents a distinct and well-defined group based on phylogenetic evidence (Miettinen et al. 2012; Binder et al. 2013; Dong et al. 2023). According to He et al. (2024), the family includes around twenty-four genera. Species of *Steccherinaceae* are widely distributed worldwide, and members of the family share several characters, including a white-rot nutritional mode, small pores or densely arranged spines, and smooth, relatively small basidiospores (Kotiranta et al. 2017; Zmitrovich 2018; Cao et al. 2021). The present phylogenetic tree (Fig. 3), inferred from the combined five-gene dataset (ITS, nLSU, mtSSU, *rpb*2, and *tef*1-α), strongly supports the segregation of the new genus *Odentinium*, which clusters with related genera *Mycorrhaphium*, *Nigroporus*, *Rhomboidia*, and *Trullella*. Morphologically, *Mycorrhaphium* differs from *Odentinium* by flabelliform pileate basidiomata with imbricate growth, a hydnoid hymenial surface, clavate basidia, and narrowly ellipsoid basidiospores (Eriksson and Ryvarden 1975). *Nigroporus* differs from *Odentinium* by having pileate to resupinate, scrupose to glabrous, azonate to concentrically zonate, grayish-blue basidiomata, a dimitic hyphal system, and allantoid to broadly ellipsoid basidiospores (Ryvarden and Johansen 1980; Li et al. 2025b). In addition, *Rhomboidia* can be distinguished from *Odentinium* by its stipitate basidiomata, a monomitic hyphal system, and broadly ellipsoid basidiospores (Xu et al. 2020). Furthermore, *Trullella* differs by having polyporoid, trematoid, or fibroporioid basidiomata; a dimitic hyphal system with clamped generative hyphae; and phaseoliform to allantoid basidiospores (Zmitrovich 2018).

From an ecological and biogeographical perspective, wood-inhabiting fungi represent an extensively studied group of *Agaricomycetes*, and species of *Polyporales* are important and widely distributed in forest ecosystems (Wang et al. 2021; Yuan et al. 2022; Zhao et al. 2023a, b, c; He et al. 2024; Hyde et al. 2024a, b). Further studies should focus on relationships between hosts and wood-inhabiting fungi. With the application of molecular phylogeny, more fungal species are expected to be reported from the Oriental realm, as wood-inhabiting fungi are cosmopolitan and particularly abundant in this region (Cui et al. 2019; Li et al. 2022; Dong et al. 2024; Yang et al. 2025). The present study aims to fill knowledge gaps regarding wood-inhabiting fungi by reporting new taxa and providing detailed morphological descriptions and phylogenetic analyses, while contributing to the enrichment of fungal diversity in Asia.

## Supplementary Material

XML Treatment for Steccherinaceae

XML Treatment for Odentinium

XML Treatment for Odentinium aurantium

XML Treatment for Odentinium cremeum

XML Treatment for Irpicaceae

XML Treatment for Efibula

XML Treatment for Efibula murina

XML Treatment for Efibula yaoshanensis

XML Treatment for Efibula zhaotongensis

XML Treatment for Phanericium

XML Treatment for Phanericium bambusacearum

## ﻿Additional information

### Use of AI

No use of AI was reported.

### Adherence to national and international regulations

All the fungal strains used in this study have been legally obtained, respecting the Convention on Biological Diversity (Rio Convention).

### Author ORCIDs

Yinglian Deng https://orcid.org/0000-0002-8220-508X

Meng Chen https://orcid.org/0009-0005-9916-8704

Kaisheng Wang https://orcid.org/0009-0007-4840-816X

Wanting Liu https://orcid.org/0009-0008-6281-8904

Daxiang Chen https://orcid.org/0009-0001-2309-1991

Shunqiang Yang https://orcid.org/0000-0003-1476-9040

Hongmin Zhou https://orcid.org/0000-0002-0724-5815

Changlin Zhao https://orcid.org/0000-0002-8668-1075

### Data availability

All of the data that support the findings of this study are available in the main text or Supplementary Information.

## Supplementary materials

Supplementary material 1 Phylogram generated from the best scoring RAxML tree Data type fas Explanation note Phylogram generated from the best scoring RAxML tree of the order *Polyporales* based on combined ITS, nLSU, mtSSU, rpb1, rpb2 and tef1-α sequenced data. Branches are labeled with maximum likelihood bootstrap values higher than 70%, and Bayesian posterior probabilities more than 0.95 respectively. https://binary.pensoft.net/file/1501738This dataset is made available under the Open Database License (http://opendatacommons.org/licenses/odbl/1.0/). The Open Database License (ODbL) is a license agreement intended to allow users to freely share, modify, and use this Dataset while maintaining this same freedom for others, provided that the original source and author(s) are credited.Yinglian Deng, Meng Chen, Kaisheng Wang, Wanting Liu, Daxiang Chen, Shunqiang Yang, Wenli Li, Hongmin Zhou, Changlin Zhao

Supplementary material 2 Divergence time estimation of families within *Polyporales* from molecular clock analysis sampling tree Data type fas Explanation note Divergence time estimation of families within *Polyporales* from molecular clock analysis sampling tree based on the combined sequence dataset of ITS, nLSU, mtSSU, rpb1, rpb2 and tef1-α. Posterior probabilities not less than 0.80 and the mean ages of each node are annotated. The 90% highest posterior densities of divergence time estimation are marked by horizontal bars. https://binary.pensoft.net/file/1501739This dataset is made available under the Open Database License (http://opendatacommons.org/licenses/odbl/1.0/). The Open Database License (ODbL) is a license agreement intended to allow users to freely share, modify, and use this Dataset while maintaining this same freedom for others, provided that the original source and author(s) are credited.Yinglian Deng, Meng Chen, Kaisheng Wang, Wanting Liu, Daxiang Chen, Shunqiang Yang, Wenli Li, Hongmin Zhou, Changlin Zhao

Supplementary material 3 Maximum Parsimony strict consensus tree illustrating the phylogeny of new genus Odentinium Data type fas Explanation note Maximum Parsimony strict consensus tree illustrating the phylogeny of new genus Odentinium and related species within *Steccherinaceae* based on ITS, nLSU, mtSSU, rpb2, and tef1-α sequences. Branches are labeled with maximum likelihood bootstrap values higher than 70%, parsimony bootstrap values higher than 50% and Bayesian posterior probabilities more than 0.95 respectively. The new species are in bold. https://binary.pensoft.net/file/1501740This dataset is made available under the Open Database License (http://opendatacommons.org/licenses/odbl/1.0/). The Open Database License (ODbL) is a license agreement intended to allow users to freely share, modify, and use this Dataset while maintaining this same freedom for others, provided that the original source and author(s) are credited.Yinglian Deng, Meng Chen, Kaisheng Wang, Wanting Liu, Daxiang Chen, Shunqiang Yang, Wenli Li, Hongmin Zhou, Changlin Zhao

Supplementary material 4 Maximum Parsimony strict consensus tree illustrating the phylogeny of *Irpicaceae* and related families Data type fas Explanation note Maximum Parsimony strict consensus tree illustrating the phylogeny of *Irpicaceae* and related families based on ITS, nLSU, mtSSU, and rpb2 sequences. Branches are labeled with maximum likelihood bootstrap values higher than 70%, parsimony bootstrap values higher than 50% and Bayesian posterior probabilities more than 0.95 respectively. The new species are in bold. https://binary.pensoft.net/file/1501741This dataset is made available under the Open Database License (http://opendatacommons.org/licenses/odbl/1.0/). The Open Database License (ODbL) is a license agreement intended to allow users to freely share, modify, and use this Dataset while maintaining this same freedom for others, provided that the original source and author(s) are credited.Yinglian Deng, Meng Chen, Kaisheng Wang, Wanting Liu, Daxiang Chen, Shunqiang Yang, Wenli Li, Hongmin Zhou, Changlin Zhao

Supplementary material 5 Maximum parsimony strict consensus tree illustrating the phylogeny of Efibula and related species Data type fasta Explanation note Maximum parsimony strict consensus tree illustrating the phylogeny of Efibula and related species based on ITS sequences. Branches are labeled with maximum likelihood bootstrap values higher than 70%, parsimony bootstrap values higher than 50% and Bayesian posterior probabilities more than 0.95 respectively. The new species are in bold. https://binary.pensoft.net/file/1501742This dataset is made available under the Open Database License (http://opendatacommons.org/licenses/odbl/1.0/). The Open Database License (ODbL) is a license agreement intended to allow users to freely share, modify, and use this Dataset while maintaining this same freedom for others, provided that the original source and author(s) are credited.Yinglian Deng, Meng Chen, Kaisheng Wang, Wanting Liu, Daxiang Chen, Shunqiang Yang, Wenli Li, Hongmin Zhou, Changlin Zhao
